# Single-cell signatures identify microenvironment factors in tumors associated with patient outcomes

**DOI:** 10.1016/j.crmeth.2024.100799

**Published:** 2024-06-17

**Authors:** Yuanqing Xue, Verena Friedl, Hongxu Ding, Christopher K. Wong, Joshua M. Stuart

**Affiliations:** 1 UC Santa Cruz Department, Biomolecular Engineering, Genomics Institute, Santa Cruz, CA, USA

**Keywords:** deconvolution, cancer genomics, The Cancer Genome Atlas, single-cell RNA-seq, survival analysis, tumor microenvironment, single-cell analysis, gene expression, systems biology, cancer systems biology

## Abstract

The cellular components of tumors and their microenvironment play pivotal roles in tumor progression, patient survival, and the response to cancer treatments. Unveiling a comprehensive cellular profile within bulk tumors via single-cell RNA sequencing (scRNA-seq) data is crucial, as it unveils intrinsic tumor cellular traits that elude identification through conventional cancer subtyping methods. Our contribution, scBeacon, is a tool that derives cell-type signatures by integrating and clustering multiple scRNA-seq datasets to extract signatures for deconvolving unrelated tumor datasets on bulk samples. Through the employment of scBeacon on the The Cancer Genome Atlas (TCGA) cohort, we find cellular and molecular attributes within specific tumor categories, many with patient outcome relevance. We developed a tumor cell-type map to visually depict the relationships among TCGA samples based on the cell-type inferences.

## Highlights

• Rank-based deconvolution improves cell-type quantification
• Single-cell RNA-seq signatures reveal cellular attributes in tumor microenvironments
• 217 cell-type signatures linked to patient outcomes in TCGA cohorts
• Tumor cell-type map illustrates cell-type composition relationships among TCGA samples

## Motivation

Analyzing bulk tumor specimens often misses crucial molecular details due to a mix of cell types within the tumor microenvironment (TME). While recent efforts have focused on the tumor immune microenvironment, less attention has been paid to the roles of other TME cells in progression and patient outcomes. Our approach leverages the growing collection of single-cell RNA-seq data to identify unique cell-type signatures across various datasets. Employing deconvolution, we analyze these signatures in the TCGA collection both on their own and together in a tumor cell-type map, revealing significant, previously unrecognized correlations with patient outcomes.

## Graphical abstract

**Figure:**
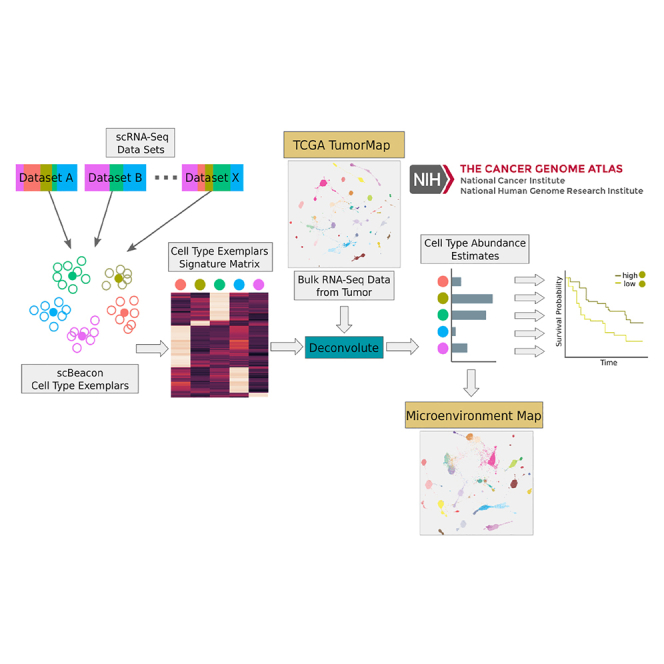

## Teaser

Xue et al. present scBeacon, an approach that leverages single-cell RNA sequencing datasets to identify 217 cell-type signatures, revealing new tumor microenvironment attributes and associations with patient outcomes across TCGA cohorts. The study introduces a tumor cell-type map to visualize these relationships, enhancing the understanding of tumor heterogeneity and potential therapeutic targets.

## Introduction

Cancer is a disease involving the interplay of many cell types.^1^ Tumor cells are surrounded by a microenvironment of various types of cells such as stromal and blood cells. Characterizing the composition and spatial arrangement of human cell types embedded in the tumor microenvironment is a relatively new direction in cancer biology research. Most notably, immune infiltration has been a focus in recent years for the implications of emerging and promising immunotherapies that have been shown to depend on the presence of certain immune cell types and states.^2^ However, studies have shown that additional cell types and molecular characters beyond immune cells play an important role in tumor character and response to treatment and patient outcomes.^3^^,^^4^^,^^5^ Therefore, it is important to detect and quantify a full profile of cell types to improve our understanding and treatment of cancer.

Characterizing the tumor cell types has been largely limited by the low number of known cell-type signatures. Most studies have focused nearly exclusively on immune-associated cell types. Leveraging cell-type signatures derived from newly available single-cell RNA sequencing (scRNA-seq) presents an opportunity to broaden the detection of cell types. scRNA-seq^6^ has transformed biological research by making it possible to determine gene expression separately for each cell in a biological sample. The technology provides a higher definition of cell types and cell states and has already expanded the catalog of known cell types.^7^ Advances in sequencing technology have facilitated an explosion of the availability of scRNA-seq datasets supported by databases such as the Single Cell Expression Atlas (SCEA)^8^ and the Human Cell Atlas.^9^ Those large databases are great resources of cell-type transcriptomes.

Over the years, there have been several bioinformatics tools developed to deconvolute bulk tumors with cell-type-specific gene expression profiles derived from scRNA-seq data. CIBERSORT(X) is a widely used cell-type deconvolution tool based on support vector regression. BSeq-SC applied scRNA-seq-derived cell-type signatures to deconvolute bulk tissues using CIBERSORT and discovered subpopulations and heterogeneity within pancreatic cell types.^10^ MuSiC deconvolutes bulk RNA-seq samples using cell-type references generated from hierarchical clustering on multi-subject scRNA-seq data using weighted non-negative least squares (NNLS)^11^; also, DeconvSeq utilizes a generalized linear model for cell-type ratio estimation,^12^ Bisque uses NNLS regression,^13^ and BayesPrism^14^ and BLADE^15^ implement a probabilistic model (multinomial) to deconvolute bulk RNA-seq data using an scRNA-seq-derived gene expression profile. These methods rely on a cell-type signature matrix from only one scRNA-seq dataset that has been pre-annotated, which limits the number of datasets used for bulk tissue deconvolution. With the increasing number of scRNA-seq datasets available and large scRNA-seq consortiums being built, strictly supervised deconvolution approaches could limit the opportunity to discover new cell types and a comprehensive characterization of bulk tissues.

Few computational resources exist that automatically extract cell-type information from scRNA-seq repositories in an unsupervised manner. SCDC^16^ leverages multiple scRNA-seq reference datasets by integrating the deconvolution results with optimized weights. UniCell^17^ is one such recent approach that uses a deep learning model trained on hundreds of fully annotated scRNA-seq datasets representing 840 cell types for comprehensive cell-type deconvolution. However, deep learning approaches can lack robustness and lead to “black box” solutions that are difficult to interpret and share. In contrast, Ecotyper^18^ used linear gene expression vectors extracted from scRNA-seq clusters that extend the original LM22 signatures into 64 immune system-related cell types used to deconvolute The Cancer Genome Atlas (TCGA) samples. Similarly, TIMEx^19^ extracted 37 immune-related cell-type signatures from a pan-cancer scRNA-seq data compendium and performed enrichment-based deconvolution on TCGA bulk tumors.

We introduce scBeacon, which infers cell types from the integration and clustering of multiple scRNA-seq datasets to provide transparent cluster “signatures” for downstream deconvolution of bulk RNA-seq specimens. It extends the work of TIMEx and Ecotyper by including additional single-cell datasets beyond cancer samples and incorporates additional cell types beyond malignant and immune system types. We introduce a non-parametric signature comparison metric that can detect related clusters across diverse datasets and merge them into a single-cell-type signature. In addition, our pipeline includes a step that tests each signature for an association with patient outcomes.

We extracted 217 cell-type signatures from SCEA, 602,359 single cells in total, and we used them to quantify cell types in bulk tumor specimens from the TCGA RNA-seq compendium. We validated the use of the signatures for deconvolution using *in silico* mixtures as well as several positive controls. We find dozens of expected and unexpected associations between cell types and tumor types in the TCGA collection, with implications for synergistic and antagonist interactions between cell types based on the co-occurrence or mutual exclusivity of cell-type groups. Some cell-type signatures were found to be significantly associated with patient outcomes in several tested tumor types, many of which are independent of published cancer subtypes and thus provide an independent measure of disease state.

To provide a comprehensive view of the relationship of all TCGA samples to each other based on their inferred microenvironment contents, we developed an interactive tumor cell-type (TCT) map that uses the inferred exemplar estimates to arrange the samples in one layout. The two-dimensional projection of TCGA samples on the tumor map^20^ revealed several unexpected cluster associations, several with implications about patient survival.

## Results

### Validation of reciprocal top-K enrichment metric for deconvolution

**Figure 1:**
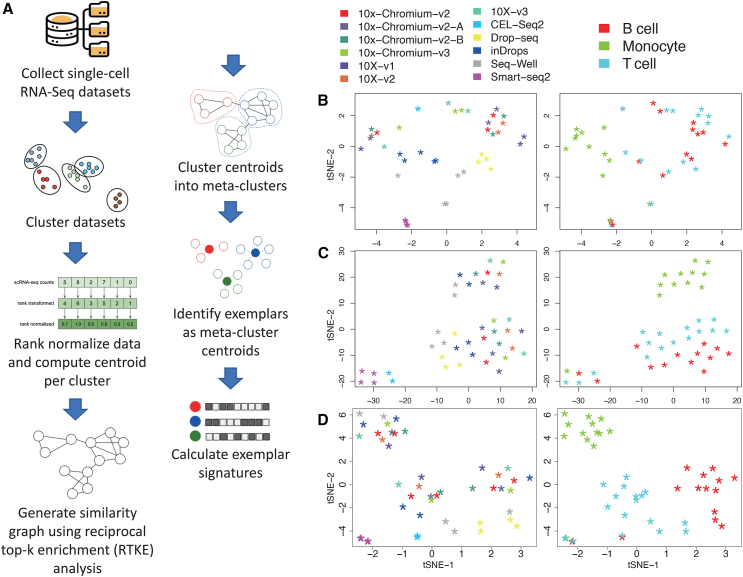
scBeacon workflow and validation (A) scBeacon workflow. Individual scRNA-seq datasets are clustered using Louvain clustering. Cluster centroids are ranked and then compared to each other using a reciprocal top-K enrichment (RTKE) metric. High-scoring cluster pairs that exceed an empirically determined threshold are retained as a graph for further clustering to identify exemplars from associated groups of meta-clusters. Exemplar centroids are computed by averaging the cluster-ranked centroids and recorded as exemplar signatures, assumed to be proxies of cell-type signatures for downstream analysis (see STAR Methods). (B‒D) tSNSE plots of PBMC scRNA-seq centroids using different transformations of the count-based data or similarity calculations between centroids. Left panel in each plot shows cells colored by single-cell sequencing technology platform (10x version 1 or 2 chemistries, green; 10x version 3 chemistry, aqua; 10x Chromium version 3 chemistry, light green; 10x Chromium version 2A chemistry, red; 10x Chromium version 2B chemistry, orange; CEL-Seq2, light blue; Drop-seq, medium blue; inDrops, dark blue; Seq-Well, purple; Smart-seq2, pink). Right panels in each plot show cells colored by cell type (T cells, light blue; B cells, red; monocytes, green). (B) Centroids represent vectors of count-based data (transcripts per million reads, TPMs). (C) Same as (B) but centroids were rank normalized. (D) Same as (C) but using the matrix of RTKE similarity metrics as input to tSNE.

The scBeacon workflow relies on *exemplar signatures*, i.e., gene expression profiles aggregated across many single cells similar enough to be clustered together, constructed from multiple clusters, from possibly multiple datasets, derived from several scRNA-seq platforms (Figure 1A; see STAR Methods). To help mitigate possible batch effects, we used signatures in which each cluster’s expression profile was rank-transformed to form a *rank centroid* before it was compared to other clusters. We created a reciprocal top-K enrichment (RTKE) metric to detect if the top-expressing genes in one cluster are also top-ranked in another, linking related clusters for a second clustering step to identify *exemplars* (see STAR Methods).

While rank transformation harmonizes data, it might weaken cell-type signals. We compared rank- and count-based centroids for deconvolution. Peripheral blood mononuclear cell (PBMC) data from multiple platforms showed that rank centroids preserved cell-type information better than count centroids (Figures 1B–1D). Except for two cases associated with smart-seq2 data (Figure 1D), ranking and RTKE enhanced the distinction among major cell types, useful for cross-platform identification.

Next, we measured the effectiveness of rank centroids for their use as exemplar signatures for deconvolving *in silico* mixtures. To this end, we created *in silico* mixtures from single-cell as well as bulk RNA-seq data that simulate immune infiltration into tumor tissue. We created *in silico* mixtures by combining several PBMC-related expression signatures together at known mixing proportions. The expression signatures were generated by taking the average of single-cell transcriptomes sampled from pre-established clusters either from the PBMC dataset or a published scRNA-seq melanoma dataset (see STAR Methods). Next, we measured the accuracy of CIBERSORT deconvolution for identifying and quantifying the PBMC cell types at the prescribed mixing proportions.

**Figure 2:**
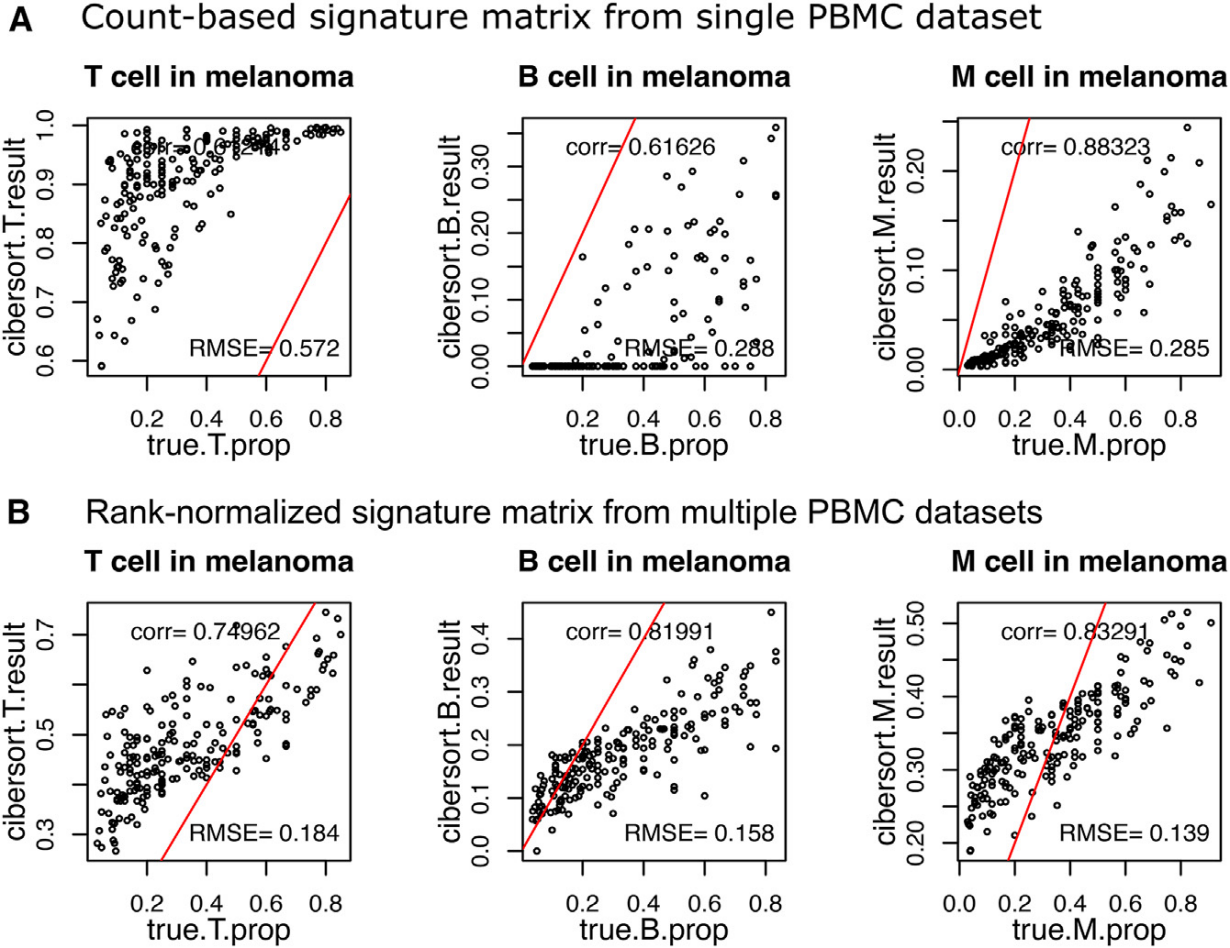
Validation of scBeacon workflow in synthetic mixtures of an scRNA-seq melanoma dataset (A) Correlation between the true mixture proportion of *in silico* mixtures from an scRNA-seq melanoma dataset to the deconvolution estimates of using a count-based signature matrix from a single PBMC scRNA-seq dataset (10X, v2). Red line marks the correct estimate (x = y). Cell-type ratios are normalized to sum up to 1. (B) Same as (A) but using a rank-normalized signature matrix from the combination of multiple PBMC scRNA-seq datasets: all PBMC datasets from Figures 1B–1D, except Smart-seq2: 10X chemistry v1–v3, CEL-Seq2, Drop-Seq, inDrops, and Seq-Well. (RMSE = root-mean-square error, corr = Pearson correlation).

We compared the use of count-based signatures to rank-based signatures for deconvolution and found that rank-based signatures provided slightly more accurate estimates of cell proportions. We used both the Pearson correlation and the root-mean-square error (RMSE) to measure the concordance between the knowns to predicted levels. For the count-based signatures, we used an immune cell-type signature matrix derived from an scRNA-seq PBMC dataset with transcripts per million reads count-based expression values to deconvolute a synthetic bulk melanoma single-cell dataset containing infiltrating immune cells (Figure 2A). For the rank-based signatures, we formed exemplar rank centroids by averaging the rank centroids of clusters found in multiple PBMC datasets (Figure 2B). While there is not a consistent trend over all three immune cell types, the deconvolution estimates using the scBeacon-derived signature matrix are generally closer to the mixed-in proportion resulting in a lower RMSE. For example, CIBERSORT tends to overestimate T cell populations when count-based signatures are used compared to rank-based ones. Rank- and count-based centroids provide comparable estimates for all cell types, with higher correlations in T and B cells and slightly lower correlation for monocytes.

In another evaluation using scRNA-seq for synthetic bulk head and neck tumors, rank-based signatures more accurately estimated B cells and monocytes than count-based ones (Figures S1A and S1B). Rank-based centroids from multiple PBMC datasets performed comparably to LM22 signatures, originally published with CIBERSORT, for deconvoluting synthetic PBMC mixtures (Figure S1). Count-based signatures overestimated T cells, while rank-based ones improved RMSE, but otherwise, the results were comparable (Figures S1C‒S1F). Systematic biases were observed, such as overestimating monocytes and underestimating B cells in the melanoma sample, likely a result of some mismatch in PBMC vs. infiltrating immune transcriptional signatures. Overall, rank-based centroids for deconvolution provided lower RMSEs and high correlations between predicted and known cell-type proportions, illustrating their effectiveness (Figures S1G and S1H).

### scBeacon clusters and signatures from EBI’s Single-Cell Expression Atlas: Building a comprehensive single-cell-derived cell-type signature library

The European Bioinformatics Institute (EBI)’s Single Cell Expression Atlas (SCEA) is a public scRNA-seq data repository that hosts datasets from published studies for six different species.^8^ For this analysis, we downloaded 62 *homo sapiens* scRNA-seq datasets available in February 2020 (Table S1). The datasets cover a wide range of healthy and diseased tissues, consisting of numerous cell types in the human body, and they were processed with different single-cell sequencing technologies.

**Figure 3:**
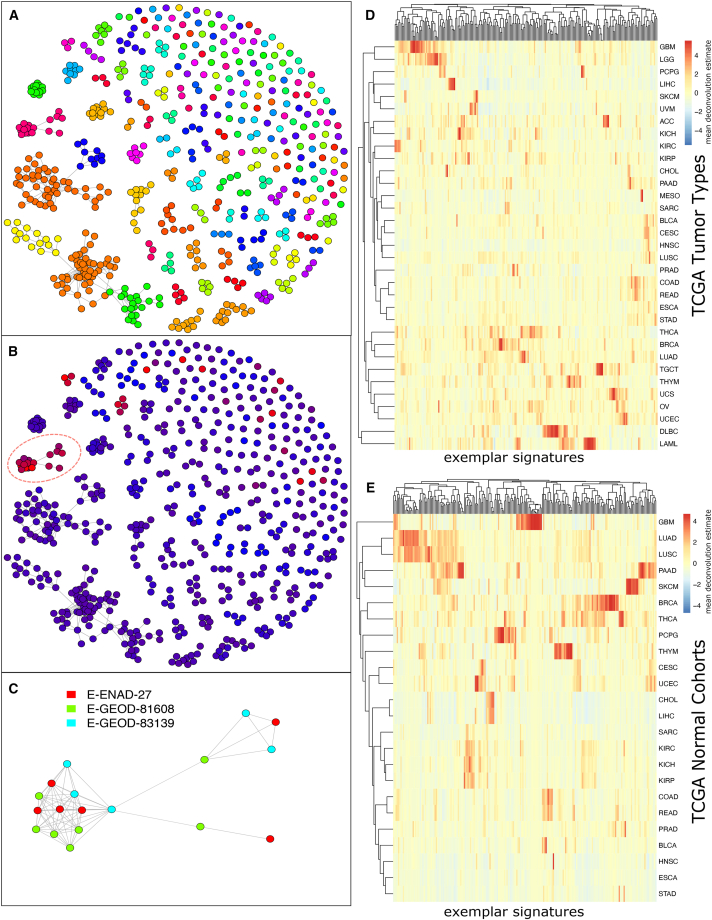
217 exemplars of cell types and states identified from RNA-seq datasets in the Single Cell Expression Atlas (SCEA) (A) Distinct cell types were identified by comparing clusters of single cells with similar expression profiles across multiple datasets. scBeacon clusters are colored by their grouping into *exemplars* representing distinct cell types/states. Nodes represent 585 clusters of single cells derived from clustering individual datasets found in the SCEA collection. To determine a non-redundant set of cell states/types from these dataset-derived clusters, clusters were connected to each other, linking clusters found in possibly separate datasets. (B) To reveal pancreas-related cell-type clusters, clusters in (A) are colored based on INS (insulin marker gene) gene expression (low expression, blue; high expression, red). Exemplar X85’s centroid (circled) had a high level of INS expression, implicating an insulin system role for its represented cell type. (C) Detailed view of the X85 exemplar illustrating it was derived from 18 different clusters (nodes) contributed by three different pancreas-related SCEA datasets (colors of the nodes), 12 clusters of which are highly mutually similar and make up the core of the exemplar. (D) CIBERSORT estimation of 217 exemplars on TCGA bulk tumor samples. Columns: 217 exemplar deconvolution estimation. Rows: averaged across the samples within each of the 33 TCGA cancer types. (E) Same as (D) but for CIBERSORT deconvolution of TCGA normals using the same set of 217 exemplars.

Clusters were extracted for each SCEA dataset, producing a total of 585 clusters. Centroids and rank centroids were calculated for each of these and used as the clusters’ signatures. Clusters were linked if their RTKE metric was above 77.39 (top 10% percentile) and then clustered into meta-clusters using the Louvain algorithm with default Seurat settings. The RTKE threshold and Louvain method were found to obtain the highest Silhouette scores out of a series of thresholds and clustering methods (K-means, hierarchical, and iGraph’s method, see STAR Methods, Figure S2). Louvain clustering produced 217 meta-clusters. Exemplar signatures were created from the average rank signatures of clusters assigned to a meta-cluster, using the top 20% of differentially ranked genes (see STAR Methods; Figure 3A). The 217 meta-clusters were annotated using author’s published annotations and an enrichment test (see STAR Methods and Table S2).

We found several examples in which multiple datasets contributed to the definitions of a single exemplar. Overall, 16 (7.4%) of the exemplars were implicated by two or more datasets (see Figure S3). Even so, the map contains many singleton exemplars—141 (65.0%), derived from a single cluster and dataset. Altering meta-clustering parameters would result in different clusters and singletons. However, we found that the chosen setting provided distinct singletons in that even those that were “close” to one another gave deconvolution results across the TCGA that were just as distinct from each other as those that were “far” apart, justifying maintaining them as separate signatures for our use (Figures S4A‒S4C). We also note that a few centroids combine clusters from different datasets that probed distinctly different human tissues. These centroids could represent a common cell type found in many tissues, as is the case with immune cell types.

We queried the scBeacon collection of exemplars to determine the extent to which they reflected distinct cell types. First, we investigated the distribution of cell types expected to be highly similar based on the expression of a particular known tissue-specific marker gene. To that end, we queried the map for all centroids with high expression of the insulin gene to identify pancreatic-associated clusters. Meta-cluster X85 contains several such pancreatic clusters (Figure 3B) that were derived from three different datasets that all assayed different states of pancreatic tissue (Figure 3C). We also queried three immune cells using marker genes, CD3E for T cells, MS4A1 for B cells, and CD14 for monocyte (Figures S4D‒S4F).

### Deconvolution of TCGA samples using scBeacon signatures

The meta-clusters from the human SCEA were further processed with the scBeacon workflow (Figure 1; see STAR Methods) to extract 3,988 genes that were differentially expressed across the meta-clusters and included in the signature matrix for use in deconvolution (Figure S5). We used CIBERSORT to deconvolute the bulk RNA-seq samples available for 33 different tumor types from TCGA^21^ using the signatures matrix derived from the 217 cell-type exemplars (Figures 3D and 3E). As expected, many cell-type signatures are undetected within most tumor samples, reflecting a degree of specificity to the signatures and their use in deconvolution. Assuming that a signature was “detected” in a sample if it had a CIBERSORT score of 0.01 or greater (i.e., it was estimated to account for 1% of the expression among all detected signatures for a particular sample after 0–1 normalization), then 83.4% of the signatures (*n* = 181) were detected in at least one sample but less than 50% of all samples. On the other hand, a small number of signatures (*n* = 2) were detected in over 90% of the samples. Finally, 10 signatures were detected at levels of 1% or less in any of the samples. These lowly-estimated signatures could represent cell types absent from the current TCGA collection among several possibilities. Still, the vast majority of the SCEA signatures (207, 95%) were detectable in at least some of the samples.

Tumor types that arise in similar tissues of the body had similar deconvolution profiles (Figure 3D). For example, the estimated cell-type profile for COAD (colon adenocarcinoma) is most similar to the estimated cell-type profile of READ (rectum adenocarcinoma). Likewise, LIHC (liver hepatocellular carcinoma) and CHOL (cholangiocarcinoma) clustered together, as well as GBM (glioblastoma multiforme) and LGG (brain lower grade glioma), and a group of squamous cell carcinomas (HNSC = head and neck squamous cell carcinoma, LUSC = lung squamous cell carcinoma, BLCA = bladder urothelial carcinoma, and CESC = cervical squamous cell carcinoma and endocervical adenocarcinoma). These results suggest that tumors arising from related tissues in the body share a similar microenvironment makeup compared to tumors arising from different tissues. Indeed, when we repeat the cell-type analysis using deconvolution on normal tissue (using the TCGA-matched normal samples), we again find that tissues cluster together based on their cell-type profiles (Figure 3E).

To confirm this result and to validate the scBeacon procedure for identifying exemplars using a positive control test case, we repeated the entire analysis using normal samples from the GTEx consortium from which exemplars were derived from the published single-nucleus RNA sequencing (snRNA-seq) dataset,^22^ and deconvolution was performed on samples from the bulk GTEx RNA-seq dataset.^23^ We found that similar tissues of related organ systems clustered together based on their GTEx exemplar deconvolution scores and that the results correlated with the results obtained with the SCEA-derived 217 signatures on the same GTEx samples (Figures S6A and S6B). For example, brain cerebellum clustered with another cerebellum, colon with small intestine with stomach, several arteries clustered together, and so on (Figures S6C and S6D). Expected cell types were again found with high deconvolution scores in GTEx tissues (Figures S6E‒S6H). Slightly more than half of the signatures in GTEx (19 out of 35; 54%) had high correlations (Pearson > 0.5) with at least one signature in scBeacon’s SCEA-derived set. Thus, we estimate another 16 signatures from GTEx could have been included to the collection of the EBI 217, consisting of a marginal increase in cell-type representation (7.4%). On the other hand, the EBI collection captured many signatures not represented in GTEx (164 out of the 217 had correlations below 0.50 for anything present among the GTEx signatures) and thus provides a 3.5-fold increase over what is represented in the GTEx collection. In summary, the meta-clustering procedure for identifying exemplars from scRNA-seq cluster signatures, as well as their use to identify them in bulk samples via deconvolution, was reproducible using a completely orthogonal dataset in a scenario where the signatures and deconvolution results were well annotated. In addition, the resulting GTEx signatures compared well to what was found and represented in the scBeacon collection based on SCEA, even though the GTEx signatures were derived from nuclei transcriptomes.

### Single-cell exemplar signatures deconvolve appropriate bulk tumors but with lower scores compared to their normal counterparts

**Figure 4:**
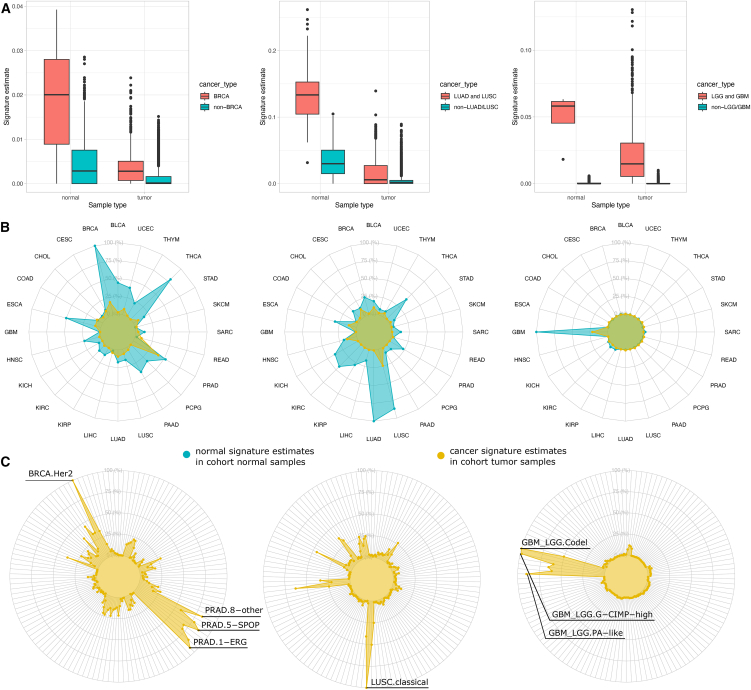
Cell-type exemplar signatures are specific to their tissue type for tumor deconvolution (A) Exemplars were selected if annotated as derived from a tissue common to a TCGA cohort. Normal and cancer exemplars were selected, either from a normal or cancer-derived cluster. Both types of exemplars show specificity to the matching tissue type in TCGA for all three tumor types inspected including breast cancer (BRCA, left panel, X10 for normal breast, X62 for breast cancer), lung cancer (LUAD and LUSC cohorts, middle panel, X41 for normal lung, X168 for lung cancer) and brain cancer (LGG and GBM, right panel, X206 for normal brain, X86 for brain cancer). The distribution of CIBERSORT estimation scores for samples within the tissue type (pink/left box in each panel) was compared to all estimations for samples outside the tissue type (blue/right box in each panel). (B) Radar plots illustrate more detail of the exemplar CIBERSORT deconvolution results in distinct tumor subsets (higher estimates correspond to outer rings) for the same cohorts as in (A) (breast cancer BRCA, left panel; lung cancers of LUSC and LUAD, middle panel; brain cancers of GBM and LGG, right panel). Each radar level shows the average CIBERSORT estimate of a cancer-related exemplar for that cancer type (yellow area) or a normal-tissue-specific exemplar for the cancer type (blue area) averaged across all TCGA samples within one of the 33 tumor types. (C) Similar to (B), but the CIBERSORT estimates of each exemplar are averaged for 132 different cancer subtypes (spokes around the circle), which group tumors based on shared molecular properties within each of the 33 tumor types.

We measured the degree to which deconvolution with exemplars, derived from a particular tissue, could “detect” the presence of a cell type in bulk tumor (or normal) samples from TCGA when using a tumor type of that same tissue. To quantify and visualize exemplar specificity, we used the CIBERSORT deconvolution results that considered all 217 exemplars to compare the estimates obtained in related to unrelated tissues. We selected three tissues—breast, lung, and brain—for which exemplars were annotated as either derived from normal or cancerous tissue. We collected the CIBERSORT estimates and aggregated them as either related to the exemplar’s tissue or unrelated. For example, X10 (myoepithelial cell of mammary gland) was used as the normal breast exemplar, while X62 (B cells from lymph node in breast carcinoma patients) was used as the cancerous breast adenocarcinoma (BRCA) exemplar since these signatures had the highest CIBERSORT scores in normal breast and cancerous breast, respectively, among all other signatures annotated as being breast related (Figure S6I). Exemplars for the other two tissues were chosen using the same criteria (Figures S6J and S6K). The 113 normal samples of the TCGA BRCA cohort showed significantly higher CIBERSORT scores for X10 than normal samples in other TCGA cohorts (*p* < 2.2e−16, Kruskal-Wallis test; Figure 4A, left panel). Similarly, the 1,104 tumor samples of the TCGA BRCA cohort showed higher scores for X62 than tumor samples in other cohorts (*p* < 2.2e−16, Kruskal-Wallis test; Figure 4A, left panel). The same trends were found for both the normal and tumor signatures when the comparisons were repeated in lung (Figure 4A, center panel) and brain (Figure 4A, right panel). Thus, exemplars annotated as derived from a specific tissue, and that have the highest match to a particular tissue in TCGA among all other exemplars annotated as derived from that tissue, also were found to be relatively specific for deconvolving that tissue (i.e., they receive the highest CIBERSORT scores among all other tissues). In summary, even when used together with signatures derived from many cell types, deconvolution of TCGA samples using the exemplars results in scores that are consistent at the tissue level.

Cancer signatures had lower CIBERSORT scores than their corresponding normal counterparts for all three of the tissue types tested (cancer boxplots in Figure 4A). This suggested that cancer signatures reflect a quantitatively lower degree of tissue specificity compared to their normal counterparts. This could be due to patient-specific factors or the loss of differentiation fidelity, among other possibilities. To further investigate, we plotted the CIBERSORT scores of both the normal and cancer samples summarized at the TCGA tumor-type level (Figure 4B) and at the level of tumor subtypes (Figure 4C). The radar plots of all three tissue types investigated reveal that, compared to the normal signatures (Figures 4B and 4C, blue radar areas), the cancer signatures (Figures 4B and 4C, yellow radar areas) have a reduced relative match to their expected tissues. For the breast and lung signatures, matches apparently similar to cell types in other tissues may explain the relative lower scores, whereas for the GBM signature, the similarity to cell types in brain-related tissue is lower without a concomitant increase in scores to cell types in non-brain tissues. In the case of the breast signature, strong matches to prostate (PRAD) cancer samples appeared to provide a better match than to breast samples when the scores were averaged. However, when the scores were averaged at the subtype level instead of at the cohort level (Figure 4C), the highest average score matched a HER2-amplified subtype of breast cancer, which represents a minor proportion of the overall BRCA samples, even though matches to several PRAD subtypes also had high scores.

Taken together, exemplar signatures had their highest relative matches in TCGA to samples obtained from the same tissues as the exemplar signatures were obtained. In addition, cancer exemplar signatures exhibited lower relative scores to their tissues on average compared to normal signatures from the same tissue. These findings suggest CIBERSORT maintains its ability to identify the presence of a cell type in a bulk RNA-seq sample using the ranked exemplar signature together with 217 total signatures. Moreover, the results indicate cancer tissue signatures may lose some of the strength of their match relative to normal tissues, which may reflect a loss of differentiation fidelity.

### Survival analysis based on deconvolution results: Some cell-type signatures align with patient outcomes in some tumor types

**Figure 5:**
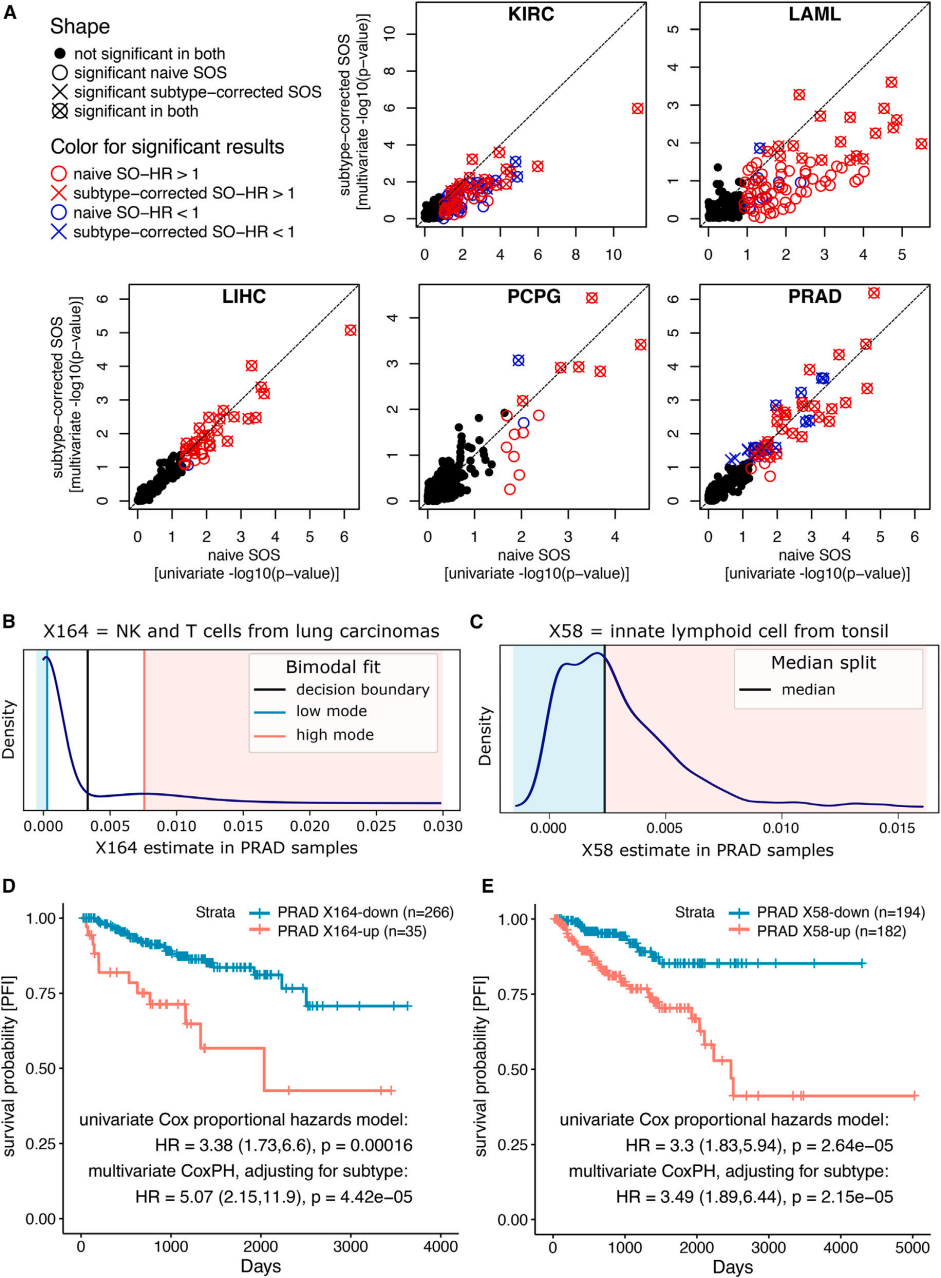
Single-cell exemplar signatures stratify patients into high- and low-risk groups in several types of cancer (A) CIBERSORT estimates for each of the 217 exemplars (circles, crosses, boxes in plots) were used to stratify patients in each cohort from low scoring to high scoring. Five cohorts had at least one signature with a significant separation (FDR < 0.25 using a Cox Proportional Harzard's (CoxPH) model). The CoxPH results of the survival separation using only the exemplar signature were plotted (“naive SOS,” x axis, log_10_ of univariate *p* value) or combined with a covariate to account for a tumor type’s published subtypes (“subtype-corrected SOS,” y axis, log_10_ of multivariate *p* value) to show those that are significant on their one (open circles), with subtype correction (crossed), or both (crossed boxes) and colored if the presence of the signature indicates a significant separation in patient outcomes (FDR < 0.25) that are either poorer (red, hazard ratio > 1) or better (blue, hazard ratio < 1) (full results in Table S3 and plotted in Figures S8 and S9). (B) For each exemplar signature in each cohort, two groups of patient samples were determined as the high and low category of the score distribution if matching a bimodal distribution. An example of such a case is shown for exemplar X164 estimated in PRAD samples where a “down group” (blue-shaded area) was distinguished from an “up group” (red-shaded area). (C) In the case where the bimodal test failed, samples were grouped into the top and bottom half using the median of an exemplar’s score. An example is shown for exemplar X58 in PRAD samples with samples below the median score defined as the “down group” (blue-shaded area) and those above as the “up group” (red-shaded area). (D) The significance by which each cell-type exemplar in each cohort separated the outcomes of the patients was measured using a Cox proportional hazards model (CoxPH) that used either the exemplar signature alone (univariate CoxPH) or combined with a covariate to account for published patient subtypes (multivariate CoxPH). The survival separation is illustrated for exemplar X164 in PRAD using a Kaplan-Meier survival plot to show that samples with estimated higher levels of the cell type represented by X164 have associated poorer outcomes. (E) Same as (D) but for a different cell-type exemplar X58 that also shows poorer outcomes when the exemplar signature is present.

We next asked whether any of the exemplars represented microenvironment determinants that indicate either better or worse outcomes for patients. To that end, we performed survival analysis separately for each cancer cohort using each of the exemplar signatures (see STAR Methods). In total, 6,944 exemplar-cohort pairs were tested, formed from the 217 exemplars tested against 32 cancer cohorts. For each exemplar-cohort pair, we grouped the patients in the cohort as either scoring high or low using the CIBERSORT estimates of the exemplar’s deconvolution proportion for each patient’s bulk tumor sample. We determined if the patient scores reflected a natural bimodal distribution (see Figure 5B for an example with signature X164 in PRAD; see STAR Methods). 2,801 exemplar-cohort pairs passed the bimodality test (*n* = 2,801). In each of these cases, the two modes were detected, and a cutoff was determined that was equidistant between the modes, dividing the samples into high- and low-scoring groups. 4,143 exemplar-cohort pairs failed the bimodality test. For these cases, the patient samples were split into two groups using the median of the score distribution as the cutoff (see Figure 5C for an example with signature X58 in PRAD).

Once two groups were determined, we asked if the presence versus absence of an exemplar’s signature implicated a difference in patient outcomes for a particular type of cancer. To that end, we calculated a signature outcome separation (SOS) measure for an exemplar applied to a TCGA cohort by fitting a Cox proportional hazards (CoPH) model using the covariate of high-/low-scoring patient group (see Figures 5D and 5E for Kaplan-Meier plots illustrating SOS for signatures X164 and X58). The significance (−log base 10) of the SOS measure was recorded as the fit of the model. Both univariate CoPH—in which only the signature was used as the predictor of outcome—and multivariate—in which an additional covariate was used that represented the previously published subtype groupings of the samples—tests were calculated. In this latter multivariate case, we refer to the SOS as the subtype-corrected SOS. A significant subtype-corrected separation would indicate an exemplar’s deconvolution score separates the patients into groups that are distinct from the established cancer subtypes, or that further separate patients within a subtype, and may be of particular biological and clinical interest.

We tested all exemplar-cohort pairs to determine if an exemplar’s signature separated the patients by their outcomes using a subtype-corrected and false discovery rate (FDR)-adjusted test (Figures S9A and S9B). We calculated SOS and subtype-corrected SOS only for pairs that had at least 10 non-zero samples in samples classified into the high-scoring category (5,931 out of 6,944). Of these, we found 5,730 cases that did not separate by outcome, across all 217 exemplars and 32 tumor types. 89 exemplars produced no outcome separation on any of the tumor types; likewise, for 27 tumor types, no exemplars were found that could separate the outcomes after accounting for the published subtypes. For example, there were 163 exemplar-tumor type pairs in which the subtype correction in the multivariate model eliminated the outcome separation detected by the univariate model. In these cases, it may be informative to investigate whether unanticipated microenvironment factors correlate with the published subtypes. However, we chose to focus on cases in which an exemplar had a clear implication on patient outcomes and that were independent of the published subtypes that we discuss next.

We found 38 exemplar-cohort pairs that had a significant subtype-corrected SOS for at least one exemplar (Figure 5A; Table 1) including four exemplars for the kidney carcinoma (KIRC) cohort, two for leukemia (LAML), four for liver (LIHC), six for the pheo- and panglioma neuroendocrine (PCPG) tumors, and 21 for prostate (PRAD). For example, four exemplars (X88, X197, X30, and X18) were found for LIHC that may reflect differentiation differences between the tumors. All four were associated with high hazard ratios, indicating poorer outcomes when the signature was detected. Moreover, the ratios were relatively unchanged in the multivariate models, indicating the exemplar-induced dichotomies of the patients are independent of the published subtypes (i.e., represent a different way of grouping the patients).

**Table 1:** All results with a subtype-corrected signature outcome separation (multivariate CoxPH model) FDR-adjusted *p* value ≤ 0.05

Tumor type	Exemplar signature	Subtype-corrected SO-HR	Naive SO-HR	Subtype OS
KIRC	X125: cortical excitatory neuron from organoids	3.64 (s)	3.70 (n)	(G)
KIRC	X184: fetal fibroblast from placenta	2.23 (S)	2.88 (N)	(G)
KIRC	X54: B cells from liver	1.84 (s)	1.68 (n)	(G)
KIRC	X145: pancreatic stellate cell	0.58 (s)	0.50 (N)	(G)
LAML	X92: astrocyte from brain	3.87 (s)	3.60 (N)	(G)
LAML	X112: stromal cell and metanephric cap from multiple tissues	3.65 (s)	2.26 (n)	(G)
LIHC	X88: oligodendrocyte precursor cell	5.32 (s)	5.40 (N)	(−)
LIHC	X197: iPSC normal culture to maintain pluripotency	3.18 (s)	3.12 (n)	(−)
LIHC	X30: spermatid and germ cells from testis	3.11 (s)	3.00 (n)	(−)
LIHC	X18: plasma cells from bone marrow	2.91 (s)	2.50 (n)	(−)
PCPG	X68: mammary epithelial cells from primary breast cancer cells and lymph node	5.93 (s)	4.59 (n)	(−)
PCPG	X205: muller cell and retinal rod cell from retinal neural layer	4.55 (s)	5.20 (n)	(−)
PCPG	X132: endothelial cells from embryonic heart	4.43 (s)	4.51 (n)	(−)
PCPG	X167: epithelial and basal cells from lung carcinomas	4.40 (s)	3.97 (−)	(−)
PCPG	X54: B cells from liver	3.98 (s)	4.62 (n)	(−)
PCPG	X39: type I pneumocyte	0.06 (s)	0.11 (−)	(−)
PRAD	X38: lung ciliated cell	6.41 (s)	4.69 (n)	(−)
PRAD	X150: acinar cell from pancreas	5.22 (S)	4.16 (n)	(−)
PRAD	X2: epithelial cells from lung bronchoalveolar carcinoma	5.13 (s)	5.36 (n)	(−)
PRAD	X164: immune from lung carcinomas	5.07 (s)	3.38 (n)	(−)
PRAD	X211: fetal hepatocytes	4.72 (s)	3.27 (−)	(−)
PRAD	X134: neurons from heart	4.02 (s)	3.52 (n)	(−)
PRAD	X122: erythroid lineage cell from multiple tissues	3.90 (s)	4.48 (n)	(−)
PRAD	X58: innate lymphoid cell from tonsil	3.49 (s)	3.30 (n)	(−)
PRAD	X44: mast cell from lung	3.47 (s)	2.90 (−)	(−)
PRAD	X145: pancreatic stellate cell	3.01 (s)	2.49 (−)	(−)
PRAD	X205: Muller cell and retinal rod cell from retinal neural layer	2.93 (s)	3.03 (n)	(−)
PRAD	X166: epithelial cells from lung carcinomas	2.60 (s)	2.33 (n)	(−)
PRAD	X196: induced neural plate border stem cells from fibroblast	2.55 (s)	2.82 (n)	(−)
PRAD	X80: acinar cell	2.54 (s)	2.93 (n)	(−)
PRAD	X132: endothelial cells from embryonic heart	2.42 (s)	2.70 (n)	(−)
PRAD	X25: EC blood from testis	2.36 (s)	2.12 (−)	(−)
PRAD	X151: alpha cells from pancreas	0.36 (s)	0.39 (n)	(−)
PRAD	X103: embryonic stem cell from H9 cell line	0.31 (s)	0.29 (n)	(−)
PRAD	X39: type I pneumocyte	0.29 (s)	0.28 (n)	(−)
PRAD	X32: peritubular myoid cells from testis	0.28 (s)	0.40 (−)	(−)
PRAD	X87: macrophages from brain	0.19 (s)	0.22 (n)	(−)
PRAD	X9: luminal epithelial cell of mammary gland	0.16 (s)	0.22 (n)	(−)

SO-HR, signature outcome hazard ratio; OS, outcome separation. The subtype-corrected SO-HR is marked with “s” if the outcome separation has a false discovery rate adjusted *p* value *p* < 0.05 and with “S” for *p* < 0.001. Similarly, the naive SO-HR is marked “n” for *p* < 0.05 and “N” for *p* < 0.001, and a tumor type for which the subtype groups show significant (*p* < 0.001) outcome separation is marked with “G.” Results for all exemplars in all tumor types are listed in Table S3.

We plotted the Benjamini-Hochberg-adjusted significance of the uncorrected and subtype-corrected SOS analysis. Most of the signatures discovered across these five tumor types were associated with poorer outcomes (red entries in Figure 5A) and no exemplars in which the outcome separation was found to be significant only after accounting for published subtypes. We note that there are three borderline significant exemplars in PRAD that may represent cases where the subtype correction does help reveal the survival separation. Other than these three exceptions in PRAD, we found that the outcome separation either remained significant (Figure 5A, crossed circles) or was no longer significant in the case that an exemplar recapitulated a separation already accounted for by the published subtypes (Figure 5A, open circles). Several cancer types (e.g., PRAD) had a linear trend near Y = X, indicating the published subtypes had little to no influence on most of the patient groupings based on signature scores. On the other hand, several tumor types (e.g., KIRC, LAML, and PCPG) had linear trends off of Y = X, revealing that subtype correction lessened the survival separation significance, suggesting many of the signature groupings are similar to the previously determined subtypes.

We found both exemplars that separate survival in a tumor-type-specific manner as well as those that separate patients by outcomes in two or more tumor types. For example, signature X132 shows an SOS in four tumor types, PRAD, KIRC, PCPG, and LGG, whereas in all those four tumor types, the detection of the signature X132 correlates with worse outcome (high SOS). The cells in signature X132 were created from four centroids from the same human dataset, of which a majority of the cells (4,568 cells out of 5,782 total, 79%) were annotated as “endothelial cells from embryonic heart.” Gene set enrichment analysis of X132 identified “GO_MUSCLE_ORGAN_MORPHOGENESIS” as the most enriched pathway from Gene Ontology. Studies have shown endothelial cells play a role in tumor microenvironment in regulating tumor initiation, progression, and metastasis,^24^^,^^25^^,^^26^ possibly reflecting a dedifferentiation mechanism to gain an immune privilege of developmental cell lineages.^27^ The SCEA contained 7 different prenatal and pediatric datasets (SCEA: E-GEOD-114530, SCEA: E-GEOD-124472, SCEA: E-HCAD-10, SCEA: E-HCAD-13, SCEA: E-HCAD-7, SCEA: E-MTAB-7381, and SCEA: E-MTAB-7407) from which 32 exemplar signature were derived by the scBeacon pipeline that included cell types originating from the liver, heart, kidney, umbilical cord blood, bone marrow, and tonsils. These signatures may implicate additional developmental associations in tumor subsets and are tabulated in the supplemental information (Table S7). Signature X112 was derived from stromal cells and metanephric cap cells of the kidney. Studies have shown that bone marrow stromal cells promote chemoresistance in acute myeloid leukemia cells^28^ and potentially negatively influence patient survival rates.

We further investigated specific exemplar-cohort pairs relevant to patient outcomes (see Table S3 documenting many others worthy of exploration). For reasons that are not clear to us, many more signatures (*n* = 22) were found to separate the patient samples of the PRAD cohort compared to other cohorts. Among these was exemplar X164 derived from lung carcinomas (dataset E-MTAB-6653), which was found to have a bimodal distribution for the PRAD samples (Figure 5A). The presence of the X164 signature was associated with poorer outcomes for PRAD patients both with and without subtype correction (Figure 5C). Our annotation pipeline associates the signature with natural killer cells and T cells of the immune system (based on PanglaoDB). Because signature X164 was derived from another cancer cohort (lung carcinoma), it is possible this immune-related signature represents a cancer-permissive state (e.g., exhausted or inhibited T cell populations). Consistent with this finding, some types of T cells, such as T_H_17 and/or T_reg_ CD4^+^ T cells, have been shown to be involved in the development or progression of prostate cancer.^29^

As another example, exemplar X58 scores did not exhibit a bimodal distribution on PRAD samples but split the samples by the median signature score (Figure 5B) yielding patient groups with different outcome classes (Figure 5D). X58 was derived from an “innate lymphoid cell” scRNA-seq dataset (SCEA: E-GEOD-70580). Studies have shown that type 2 innate lymphoid cells are enriched in prostate cancer,^30^ which produce interleukin (IL)-4 and IL-13, and are known to regulate tumor microenvironment and promote cancer proliferation.^31^^,^^32^

### Pan-cancer clustering on a tumor cell-type map using all cell-type exemplar signatures

We projected the TCGA samples onto a two-dimensional landscape, using the estimates of all 217 cell types as input to the UCSC TumorMap,^20^ producing an interactive TCT map available online at bit.ly/TCTmap_217exemplars. We clustered the samples using a spatial hierarchical method called hdbscan^33^ to identify 50 TCT clusters, out of which 35 were “pan-cancer,” consisting of at least two tumor types. If a tumor type had at least five samples in multiple clusters, an outcome analysis was performed between the main cluster of that tumor type and the smaller minor cluster(s) (see STAR Methods, Table S4).

**Figure 6:**
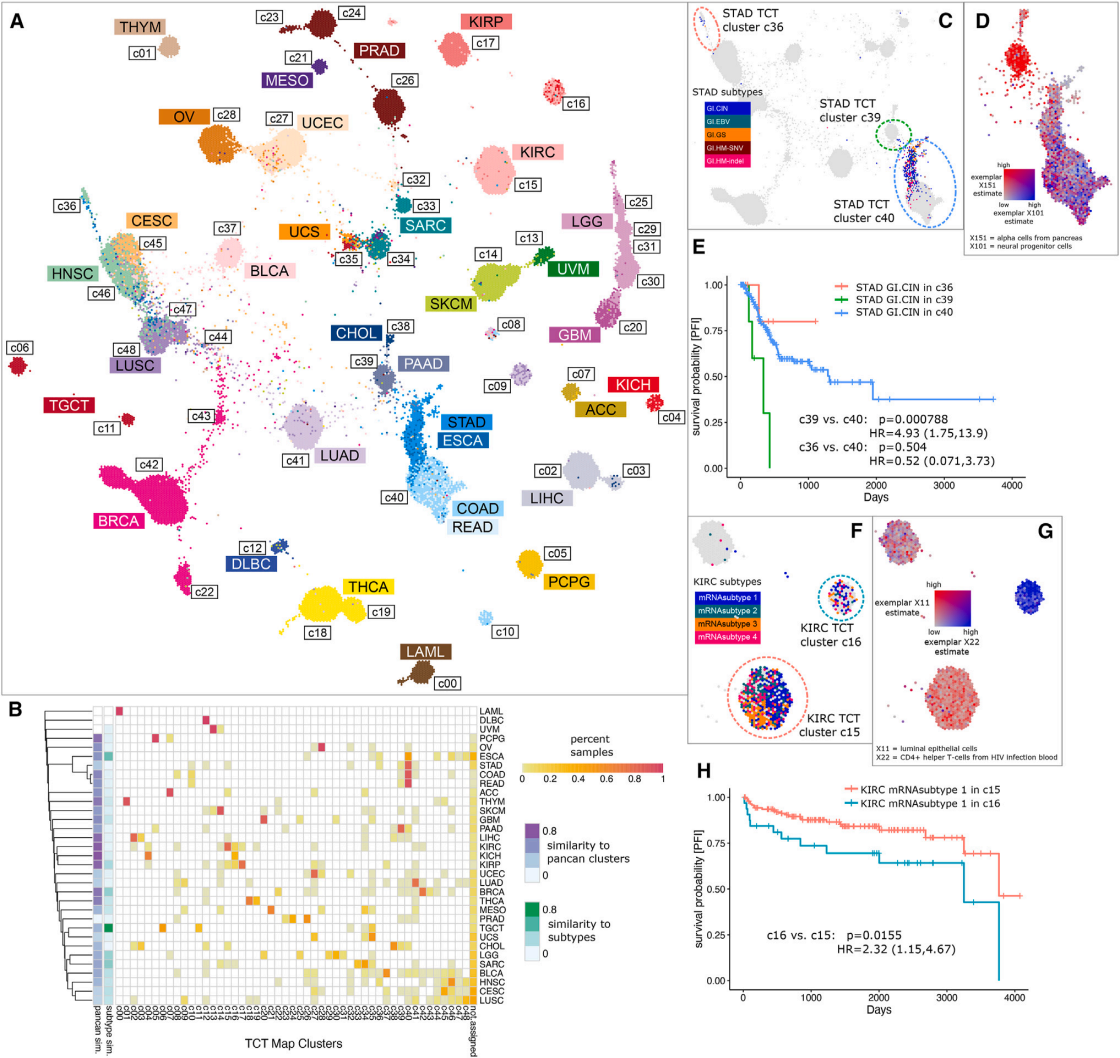
Tumor cell-type (TCT) map (A) TCT map colored by TCGA tumor type. (B) Clustering of tumor types into clusters and the similarity (adjusted Rand index) of the clusters to the clustering solution derived in TCGA PanCanAtlas^21^ as well as the grouping of samples into tumor subtypes. (C) STAD samples, colored by STAD subtypes. (D) Colors show the most differential signatures in STAD GI.CIN samples between cluster c39 and c40: exemplar X151 (alpha cells from pancreas) and exemplar X101 (neural progenitor cells). (E) Survival of STAD subtype GI.CIN samples based on their clusters. Cox proportional hazard's (CoxPH) models were used to calculate p-values between two clusters of the same cancer type including subtype information as a covariate to account for subtype imbalances. (F) KIRC samples, colored by KIRC subtypes. (G) Colors show the most differential signatures in KIRC mRNA subtype 1 samples between cluster c15 and c16: exemplar X11 (luminal epithelial cells of mammary gland) and exemplar X22 (CD4-positive helper T cells from HIV infection blood). (H) Survival of KIRC mRNA subtype 1 samples in clusters. Statistical analysis to calculate *p* values was performed as described in part (E).

Most samples cluster by their tumor type (Figure 6A) as expected due to the cell-of-origin signal in mRNA-seq data.^21^ Even so, some exceptions were observed in which TCT clusters revealed unanticipated divisions. Interestingly, the TCT clusters corresponded highly to PanCanAtlas groupings relative to published subtypes in many cases. We compared the TCT clusters with previous subtypes quantitatively for each tumor type (Figure 6B). STAD had low similarity to both PanCanAtlas clustering solution and published subtypes. The STAD samples were oriented into three main TCT clusters—c36, c39, and c40 (Figure 6D). Each of the three clusters contained a mixture of the published STAD subtypes. Thus, STAD as well as 6 other tumor types (i.e., CESC, LUSC, PRAD, LUAD, UCEC, and UCS) reveal TCT factors correlated with exceptions to the general rule in which tumors cluster with others primarily of their same tissue of origin. We discuss several examples of “splitting” or “merging” of TCT clusters relative to the expected pattern.

We found several cases in which the TCT map split a published subtype into multiple new clusters and cases where the map merged samples of previously separated published subtypes into a single new cluster. A splitting pattern was found for the STAD cohort where samples annotated originally by TCGA as copy number unstable (i.e., the STAD-GI.CIN subtype) were divided into three TCT clusters and revealed a survival difference among the patients for two of these clusters (c39 and c40, Figure 6D). For example, cluster c39 patients have significantly lower progression-free interval (PFI) survival rates compared to c40 patients, and both c40 and c39 patients have lower PFI survival probability compared to c36 patients. c39 had a higher signal from exemplar X151 (alpha cells from the pancreas), and c40 had a higher signal from exemplar X101 (neural progenitor cells), compared to c39. We suspect that the alpha cell exemplar reflects the enteroendocrine signal in STAD samples due to the current lack of a stomach enteroendocrine exemplar in the scBeacon collection as endocrine cells are found throughout the gastrointestinal tract.^34^^,^^35^ The association of X101 with c40 suggests the enteric nervous system (ENS) marks distinct tumor microenvironments, which is supported by work showing the ENS plays an essential role in regulating both the stem cell niche and the tumor microenvironment in many organs.^36^

We found examples in which the TCT map clustered merged together samples belonging to different previously published subtypes. For example, TCT clusters c15 and c16 contain a mix of published KIRC subtypes (Figures 6F–6H). TCT cluster c15 had higher signal from exemplar X11(luminal epithelial cells), while cluster c16 had higher X22 signal (CD4^+^ helper T cells from HIV infection blood). For KIRC mRNA subtype 1 samples between cluster c15 and c16, samples in c15 had better survival rates compared to c16, possibly due to the role that tumor epithelia play in regulating immunotherapy outcomes and molecular components in tumor microenvironment.^37^ In Zhang et al., the authors found KIRC-TCGA samples with high estimated fraction of CD8^+^ T cells have lower survival probability than samples with high estimated endothelial cells, consistent with the dissimilar survival trend we observed for TCT cluster c15 versus c16.

Visual inspection of the TCT map revealed additional examples of groupings that go against the expected trends (cancer types or their subtypes clustering) that may suggest microenvironment factors associated with tumor state. For example, the TCT divided some of the lung cancers into two distinct clusters (c09 versus c41) with both clusters having equal representation from the major subtypes (LUSC and LUAD). The division appeared to separate potentially different lineages with c09 showing higher levels of X38 (lung ciliated cell) and X41 (transformed epithelial cell from lung) compared to those with higher levels in c41 such as X167 (epithelial and basal cells from lung carcinomas) and X46 (type II pneumocytes). As another example, uterine carcinomas (UCEC) had an interesting pattern in the TCT. Among the copy number high UCEC subtype samples, several clustered with the serous ovarian tumors in c28 (*n* = 30), while others clustered into the c27 group (*n* = 114). The microenvironment factors that underlie the UCEC copy number high distinctions are complicated to interpret as both the high signatures in c28, such as X59 (neurons in the neocortex), and the high signatures in c27, such as X43 (B cell from lung), were annotated with lower confidence. Other UCEC samples cluster with sarcomas into TCT cluster c32, distinguished by high levels of signature X108 (keratinocytes/suprabasal cells of esophagus) and low levels of the well-annotated signature X168 (basal cells from lung carcinomas). More precise cell-type signatures may be needed to understand the major determinants of the UCEC divisions by the TCT. On the other hand, some of the uterine sarcomas (UCSs) clustered with the UCEC samples into c27 (*n* = 13) instead of the main cluster (*n* = 14) with immune-related signatures correlated with the division, e.g., with X22 (CD4-positive helper T cell from HIV infection blood) higher in the UCEC cluster compared to epithelial cells (X132 and X117) that are higher in the other cluster. Finally, the TCT map divided some of the prostate (PRAD) samples into two clusters that were not subtype related, with some PRAD samples clustering into c24 (*n* = 199), and others clustering with samples in c26 (*n* = 296), with higher levels in c24 associated with exemplars X152 (acinar cell from pancreas) and X156 (CD8-positive T-lymphocytes from influenza patients), reflecting a lineage difference (e.g., involving the secretory glands) and/or a variation in the immune components underlying the disease. Thus, the TCT map revealed commonalities among tumors previously considered to have distinct molecular profiles.

## Discussion

There is ever-growing evidence that the cell types present in a tumor’s microenvironment influence the outcome of a cancer patient.^1^ In recent years, since single-cell sequencing became available, the characterization of various cell types in the human body has improved immensely.^38^^,^^39^^,^^40^ A growing number of public single-cell sequencing datasets provide a more accurate and comprehensive definition of the human cell type repertoire. However, there are still challenges to efficiently integrate and analyze those datasets together. First, due to the high level of technical noise and systematic differences between sequencing platforms, simple concatenation could result in batch effects that become the dominant variance rather than biology. Batch effects have been shown to cause an increased number of false positives in downstream analyses.^41^ To reduce the chance of false discoveries, integration of multiple datasets must eliminate batch effects.^42^ Whole reference atlas initiatives such as the Human Cell Atlas started collaborative projects to integrate as many datasets as possible to create a whole human cell-type map, and the data integration process for this task should not only be able to handle batch effects well but also be computationally efficient and fast while ingesting and integrating datasets.

We introduced a scRNA-seq pipeline called scBeacon that clusters and integrates datasets to identify single-cell signatures useful for the deconvolution of bulk cancer samples. Unsupervised clustering of full transcriptome data has been used to identify subsets of related samples or genes for years since the establishment of DNA microarrays.^43^^,^^44^ Since then, clustering has only increased in importance for the analysis of bulk and later scRNA-seq datasets.^45^ Computational algorithms leverage an ever increasing number of samples of scRNA-seq datasets using approaches like community detection^46^ and later deep learning autoencoders.^47^ In our approach, we assume many of the clusters represent a collection of cells with highly similar transcriptomes that concentrate distinct cell types. Given this assumption, “marker genes” of a cell type/state or lineage may be approximated with the cluster centroids. Our pipeline infers cell types using multiple datasets by using an enrichment-based test to determine when clusters from different scRNA-seq datasets are highly similar.

We validated scBeacon’s deconvolution using *in silico* mixtures from single-cell and sorted bulk RNA-seq data. We used EBI’s SCEA as a database to create a comprehensive set of cell-type signatures with an enrichment-based similarity test, the RTKE test. We used the resulting 217 signatures for the deconvolution of 33 different cancer types from TCGA. Many of the cell-type signatures are found to be correlated to patient outcomes in single tumor types, some also over multiple tumor types.

Several methods have been created to help biologists search these collections to find cell types of interest. Scmap^48^ implemented a fast approximate k-nearest-neighbor search with cosine distance to project cells in scRNA-seq datasets to reference databases. CellBlast^49^ built a robust data query method in an scRNA-seq database based on a neural network-based generative model and a customized cell-to-cell similarity metric. CellAtlasSearch^50^ used locality-sensitive hashing Hamming distance for bulk and single-cell RNA-seq data processing and query. We found the RTKE test to be robust in the comparison of cluster centroids across datasets and scRNA-seq platforms.

We found that the use of rank-based cell-type signatures for the deconvolution of bulk RNA-seq data compared to count-based cell-type signatures is effective for forming signatures from multiple data sources. The rank normalization and combination of multiple datasets did not impact the accuracy of deconvolution and sometimes even improved the inference. Thus, our rank-based approach offers a promising and simple strategy for the ongoing derivation of a comprehensive set of cell-type signatures from an expanding collection of scRNA-seq datasets.

Validation of the scBeacon approach using the GTEx consortium data further affirmed its robustness and accuracy in identifying cell-type signatures and their application in deconvolving bulk RNA-seq datasets. By leveraging the orthogonal dataset published by the GTEx consortium containing snRNA-seq-derived signatures and subsequent deconvolution of GTEx bulk RNA-seq samples, we demonstrated that deconvolution with scBeacon-derived signatures for GTEx effectively grouped similar tissues, thereby underscoring its utility across diverse biological datasets. Notably, tissues from related organ systems such as the brain, gastrointestinal tract, and vascular structures exhibited coherent clustering, which is indicative of the tool’s precision in capturing organ-specific cellular compositions. Moreover, the comparison of the scBeacon-derived signatures from the SCEA with the 35 obtained from GTEx revealed significant overlaps, with more than half of the GTEx signatures showing a high correlation with the SCEA-derived set. The majority of SCEA signatures were unique compared to those in GTEx, indicating a broader scope of cellular diversity captured by the SCEA dataset, reinforcing the capability of scBeacon to provide a detailed and expansive view of cellular landscapes across different conditions and tissues, which is crucial for understanding complex biological systems and their underlying mechanisms in health and disease.

In summary, we provide a comprehensive collection of cell-type signatures based on the preprocessing of a large amount of scRNA-seq data, strategies for identifying and merging signatures across datasets even from different platforms using rank-based centroids, a graph-based meta-clustering approach, and an enrichment-based cluster comparison metric. We provide annotations for all of the discovered 217 signatures and document survival associations for 33 exemplar signatures in 5 tumor types. We have made available an interactive map of all TCGA tumors based on their TCT content. We found evidence for both merging pre-established subtypes into common TCT clusters as well as splitting samples of one subtype into multiple TCT clusters. We found several examples in which regrouping samples, either using individual signatures on a single tumor cohort or using all signatures in a pan-cancer TCT clustering, revealed unexpected outcome implications.

### Limitations of the study

The interpretation of the deconvolution results has challenges. When a cell type is detected in a cancer sample, it may be due to the cell type being present in the tumor microenvironment. However, another possibility is that the tumor cells themselves have acquired certain characteristics of other cell types, which are ascribed to a particular cell type by the deconvolution method. Yet another possibility is that the usage of an incomplete reference might influence the deconvolution estimate to detect the most closely related cell type when the actual cell type is not included in the signature matrix. In addition, the annotation of the established collection of cell-type signatures is challenging since only a subset of the clusters of a dataset may have reliable annotations either assigned by the authors or inferred by computational methods like those presented in this study. Finally, the granularity of our cell-type signatures may have an effect on the downstream analysis. Some datasets in our database are represented completely by just one cell-type signature. This happens because all cells in the dataset are from a specific cell type and are very similar to each other compared to other datasets. Nevertheless, a more fine-grained cell-type definition might be desirable in some cases, and a hierarchical definition of cell types and cell-type signatures might be a solution to this issue.

TCGA does not contain an exhaustive representation of all tissues and cell types in the body. Indeed, it has a limited set of cancer types. Thus, we expect many cell types to be absent from the TCGA collection. The fact that some signatures are not found when deconvolving may either be the exclusion of certain types of cells in cancer tissues in the biased TCGA set or “odd” cell types found in scRNA-seq data that are not present in bulks samples (although the latter is hard to rule out as we did not analyze a comprehensive set of bulk tissue data). As the collection of signatures grows, there will concomitantly be increases in the number of signatures that fail to be detected in any analyzed set of tissues. However, at this stage, such extra cell types have not proven to be detrimental to the deconvolution or downstream analyses in any tangible way.

## STAR★Methods

### Key resources table

**Table:** 

REAGENT or RESOURCE	SOURCE	IDENTIFIER
**Deposited data**		

Single Cell Expression Atlas (SCEA) scRNA-seq data	Papatheodorou et al.^8^	see Table S1
TCGA bulk RNA-seq data	Hoadley et al.^21^	see Table S1
scRNA-Seq data from a human head and neck cancer dataset	Puram et al.^51^	GSE103322
human melanoma dataset	Tirosh et al.^52^	GSE72056
MSigDB gene set collection	Liberzon et al.^53^	https://www.gsea-msigdb.org/gsea/msigdb/
scRNA-Seq PBMC datasets	Ding et al.^54^	see Table S1
scRNA-Seq PBMC	10X genomics	https://www.10xgenomics.com/datasets/10-k-pbm-cs-from-a-healthy-donor-v-3-chemistry-3-standard-3-0-0
Bulk RNA-Seq data for each of melanoma cell lines	Pawlikowski et al.^55^	see Table S1

**Software and algorithms**		

CIBERSORT	Chen et al.^56^	https://cibersortx.stanford.edu/
Tumor Cell Type (TCT) UCSC TumorMap	Newton et al.^20^	bit.ly/TCTmap_217exemplars
Seurat	Stuart et al.^57^	https://satijalab.org/seurat/
PanglaoDB	Savić et al.^58^	https://panglaodb.se/
Harmonizome	Rouillard et al.^59^	https://maayanlab.cloud/Harmonizome/
Original code for data analysis	This paper	https://doi.org/10.6084/m9.figshare.25814632.v1

### Resource availability

#### Lead contact

Further information and requests for resources and reagents should be directed to and will be fulfilled by the lead contact, Josh Stuart (jstuart@ucsc.edu)

#### Materials availability

This study did not generate new unique reagents.

#### Data and code availability

• This paper analyzes existing, publicly available data. The accession numbers for the datasets are listed in the key resources table.
• All original code has been deposited at Figshare and is publicly available as of the date of publication. DOI is listed in the key resources table.
• Any additional information required to reanalyze the data reported in this paper is available from the lead contact upon request.

### Method details

#### EBI single-cell expression atlas

##### Datasets and cluster centroids

The Single Cell Expression Atlas (SCEA), a part of EMBL-EBI’s Expression Atlas, is a public single-cell RNA sequencing data consortium that hosts datasets from published studies for six species.^8^ For this analysis, we downloaded the 62 homo sapiens single-cell RNA sequencing datasets available in February 2020 (Table S1). The datasets come from a wide range of healthy and diseased tissues, consisting of numerous cell types in the human body. The 62 datasets were sequenced using different single-cell RNA sequencing techniques, such as 10X Genomics platform, smart-seq, drop-seq etc.

##### scBeacon - Exemplar signature derivation

The scBeacon workflow is shown in Figure 1A. Starting from a compendium of scRNA-Seq datasets, the cells in each dataset are clustered using the Louvain algorithm.^46^ We found louvain clustering had the best performance regarding speed and memory efficiency on different environments (dgtMatrix in R, pandas data frame in python) compared to various other clustering algorithms, e.g., k-means and hierarchical clustering (Figures S2A‒S2E).

We used cell-wise rank normalization to reduce any possible batch effects that would be introduced by integrating clusters across different datasets. For each cell cluster, a centroid was computed by taking the average rank of a gene across all the cells in the cluster, resulting in a rank average for each gene. We found that rank normalized centroids were accurate and robust representations of single-cell clusters. First, we found that rank centroids accurately preserved biological information using the MOCA (Mouse Organogenesis Cell Atlas) dataset^51^ as a test case. Centroids “islands” in different colors were found to represent unique cell types in MOCA (Figure S7A) and the developmental trajectory was well preserved according to the annotated murine developmental stages (Figure S7B). Second, we found that generating rank normalized centroids from 50 cells is robust to represent a cluster based on subsampling cells and finding that the Spearman correlation between the centroid derived from a random subset and its corresponding centroid from the complete data saturates at 50 (Figure S7C).

In order to group centroids by unique cell types, we used the reciprocal top-k enrichment (RTKE) method introduced in the Biological Process Activity manuscript by Ding et al.^52^ After rank-normalization, the top 10 percent of the genes in a centroid were used to perform RTKE and the enrichment scores were used as similarity scores to compare all centroids to each other. We found that other choices for the top k genes, ranging from 5% up to 40%, yielded highly similar results as using the top 10% of genes (Figure S2J).

To cluster the cluster centroids into meta-clusters that include similar cell types, we compute the empirical distribution of similarity scores. We set a threshold for centroids as 0.006 upper quantile of the empirical distribution and then use the louvain clustering to define meta clusters. Each meta-cluster is considered to represent a cell type, and it can be made of multiple centroids from one or multiple datasets or be a unique cluster from just one dataset. The 0.006 top quantile is selected by screening through thresholds ranging from 0 up to 0.1, the clustering results are evaluated using Silhouette scores, when threshold is 0.006, Louvain clustering reaches the highest Silhouette score (Figure S2K).

The meta-cluster centroids, also called exemplars, are used as cell-type signatures. To obtain cell-type signatures for tumor deconvolution, we first constructed a differential gene expression matrix. For each cell type, we identify a unique set of genes that distinguishes it from other signatures. First, we compute the average expression of each gene in the 217 cell types. For each gene, we subtract the average expression value of the highest-expressing cell type and the second-highest expressing cell type. This strategy ensures that only genes expressed distinctly high in each cell type are included in the signature matrix, which is key for subsequent analyses as overlap in gene signatures between cell types can complicate deconvolution results. The 20% most differentially expressed genes are chosen as signature genes and this subset matrix was used as the signature matrix as the input for CIBERSORT. Figure S5 shows a heatmap of the 3988 unique genes that were used in at least one of the 217 cell type signatures. We used this signature matrix in bulk tumor deconvolution.

##### Annotating the SCEA signatures using pathway enrichment

To better understand the biological features of SCEA signatures, we use GSEA enrichment analysis to test for both enriched cell types and pathways by using a combination of gene sets from PanglaoDB,^55^ Harmonizome,^54^ and the cell type pathways from MSigDB (C8). To maintain specificity as well as robustness for the enrichment analysis, we retained gene sets that had more than 50 genes and less than 100 genes. This resulted in a collection of 5398 gene sets in total – 178 from PanglaoDB, 84 from Harmonizome, 4436 from MSigDB GO genesets and 700 from MSigDB cell type genesets(Table S6). For each signature, we used GSEA to score and rank all of the gene sets in the collection. The top five ranking gene sets for each cluster was recorded in an annotation table (Table S2). We also used cell-level annotations published in the manuscripts that described the dataset from which a cluster was derived and prioritized using these author-provided annotations to label a cluster centroid wherever it was available. If multiple annotations were present among the cells in a cluster, a summary annotation “short name” was created. Manual inspection of the cases where author annotations and PangloDB-inferred annotations were both available revealed a high concordance between the independently derived annotations (see Table S2). In the absence of an author-derived annotation, a “short name” was created by summarizing the top ranking gene sets for the associated signature.

#### Deconvoluting cell types in bulk tumors

The exemplar cell-type signatures generated from the scBeacon workflow were used for deconvolution of cancer bulk RNA-Seq data, in which each signature’s contribution to the mixture was estimated. We used the Cibersort deconvolution method,^60^ which performed well in the DREAM deconvolution competition.^56^ We ran Cibersort with parameters: perm = 100, QN = FALSE, absolute = TRUE, abs_method = 'no.sumto1'.

We used rank-normalized cell type signatures in CIBERSORT to deconvolute TCGA bulk tumors. Compared to cell type signatures derived from count-based expression values, rank-normalized signatures outperformed count-based signatures in bulk tumor deconvolution, which is commonly used in other deconvolution approaches. This was validated by our validation analysis using synthetic bulk samples.

In this study, we used the TCGA collection as the bulk tumor data for deconvolution. We downloaded the counts per tumor type data from Xena,^57^ which represents The Cancer Genome Atlas (TCGA) gene expression HTSeq counts data originally provided by the NCI’s Genomic Data Commons. We normalized the count data to TPM (transcripts per million reads).

#### Validation experiments

##### Tissue-specificity evaluation

To determine the extent to which the 217 cell type signatures reflect their specific tissues of origin when used in deconvolution, we identified a collection of breast, lung, and neural signatures from normal tissues, cancer tissues, and evaluated their tissue specificity in TCGA samples using the highest estimated signatures.

We first selected datasets that contain the cell type of interest, then collect and evaluated the cell type signatures that come from those datasets. Here are the signatures we collected that represent specific tissues. Breast normal tissue signatures: X7, X8, X9, X10, X11, X12; Breast cancer tissue signatures: X62, X63, X64, X65, X66, X67, X68; Lung normal tissue signatures: X38, X39, X40, X41, X42, X43, X44, X45, X46, X47; Lung cancer tissue signatures: X1, X2, X114, X120, X163, X164, X165, X166, X167, X168, X169, X170; Neuron normal tissue signatures: X125, X199, X200, X201, X202, X203, X204, X205, X206; Neuron cancer tissue signatures: X5, X86, X87, X88, X89, X90, X91, X92, X93.

To pick the most representative cell type signatures, we computed the average estimation of the collection of signatures in their specific tissues in TCGA samples, and select the top estimated signatures to compare within the entire TCGA cohort (Figure S6). Then we made radar plot that shows the CIBERSORT estimation of the signatured that comes from one tissue, both normal and cancer state. The radar plot shows the normal tissue signatures has higher tissue-specificity compared to its cancer tissue signature (Figure 4).

##### *In silico* immune infiltration evaluation

We created different types of in silico cell type mixtures simulating immune infiltration in cancer tissue in order to validate the 217 cell type signatures for deconvolution. We created 200 in silico mixtures from scRNA-Seq data from a human head and neck cancer dataset^53^ (GSE103322), human melanoma dataset^58^ (GSE72056), and bulk RNA-Seq data for each of 6 different melanoma cell lines.^59^ The centroid of all scRNA-Seq tumor cells or bulk RNA-Seq cell lines in each dataset was used to represent the tumor component of the mixture. The tumor component was randomly assigned a mixture percentage between 50 and 90%. The rest of the mixture was randomly distributed between immune and microenvironment cell-type centroids in integer-valued percentages: B cells, dendritic cells, NK cells, endothelial cells, fibroblasts, macrophages, mast cells, myocytes, and T cells.

For the melanoma cell lines dataset, the immune cell types were purified from blood using marker genes in a vaccination study.^61^ We take the average of the 2 patients at time point t_0_ (before vaccination) to represent pure cell type references. For both datasets, the expression data were reduced to the overlapping genes between the two datasets and quantile normalized to remove batch effects and enable mixing.

To validate this approach, we used scRNA-Seq PBMC (peripheral blood mononuclear cell) datasets from different sequencing platforms.^62^ PBMCs consist mainly of monocytes, B cells, and T cells, with other minor fractions of dendritic cells, NK cells, and macrophages.^63^ We created cell-type signatures from scRNA-Seq PBMC datasets^62^ from various single-cell sequencing technologies, e.g., 10X Chromium, CEL-Seq2, Drop-Seq, inDrops, Seq-Well. Additional PBMC datasets were downloaded from the 10X Genomics website, Chromium demonstration data^64^.

(1) Dataset by Cell Ranger 1.1.0, published on July 31, 2016
(2) 10X-v2: 8k PBMCs from a Healthy Donor, Platform: 10XGenomics v2 chemistry, Single Cell Gene Expression Dataset by Cell Ranger 2.1.0, published on November 8, 2017
(3) 10X-v3: 10k PBMCs from a Healthy Donor, Platform: 10XGenomics v3 chemistry, Single Cell Gene Expression Dataset by Cell Ranger 3.0.0, published on November 19, 2018

We clustered each dataset using the Louvain algorithm and assigned three main clusters to monocytes, B cells, and T cells using the expression of established marker genes (CD3E for T cells, MS4A1 for B cells, and CD14 for monocytes). We calculated centroid for each cell type and generated a signature matrix for each dataset.

We performed three different deconvolution approaches with the signatures to determine if ranking the centroids and combining the signatures produced accurate deconvolution results. First, the log-transformed, count-based TPM (transcripts per million reads)-normalized centroids from the 10X-v2 dataset alone were used as the signature matrix in deconvolution. Second, the rank-normalized centroids from the 10X-v2 dataset were used on their own as the signature matrix for deconvolution. Finally, the rank-normalized centroids were combined with all PBMC scRNA-Seq datasets and used as a combined signature matrix in deconvolution.

#### Tumor cell-type (TCT) map of pancancer connections

##### Building the map

The two-dimensional layout of the Tumor Microenvironment (TCT) map was created by providing the matrix of the 217 exemplar CIBERSORT estimates for each of the 11,057 TCGA samples to the DrL layout engine of the UCSC TumorMap tool.^20^

##### Clustering the samples on the TCT map

The samples on the TCT map were clustered by their two-dimensional coordinates using hdbscan, a spatial hierarchical clustering method,^33^ with a minimum cluster size of 20. This resulted in 49 sample clusters (Figure S10A). Additionally, 1,277 samples were not assigned a TCT map cluster.

To measure the difference of the resulting clustering solution and previously published clusterings of the tumor samples, we first measured the similarity between the spatial TCT map clusters and the grouping by disease subtype using the adjusted rand index. Additionally, we measured the similarity to the PancanAtlas mRNA-based TumorMap.^21^ This TumorMap provides a similar comprehensive look at the same set of TCGA samples, and it is based on mRNA data, which is also the basis of our exemplar estimates. Therefore, we can now determine if any grouping we find on the TCT map is only a recapitulation of known subtype biology or gene expression, or if it is newly determined by our exemplar estimates.

We applied the same spatial hdbscan clustering method to the PancanAtlas mRNA TumorMap with a minimum cluster size of 50 samples in order to reach a similar number of resulting clusters (Figure S10B). The samples on the PancanAtlas TumorMap were assigned to 41 clusters and 1,123 samples were not assigned a cluster. We then measured the similarity of the two spatial clustering solutions using the adjusted rand index.

### Quantification and statistical analysis

#### Signature-cohort bimodality test

After obtaining the CIBERSORT deconvolution results on TCGA cancer samples, we analyze if the presence of the cell-type signatures in tumors correlates with the survival outcomes of patients. First, we define patient groups based on how much a signature is detected in the patients’ tumor samples. For each signature in each tumor type, samples that have a relatively high proportion of the signature detected are defined as “patients-up group” and samples that have a relatively lower proportion of the signature detected are defined as “patients-down group”.

To formalize this separation of samples in the deconvolution results, we applied a bimodality test for each signature, based on the student-t distribution^65^ implemented in the t-Student Mixture Models Module (SMM) library^66^ in python. It models data by a mixture of t-Student distributions, estimating the parameters with Expectation Maximization, and uses the Bayesian information criterion(BIC) to decide whether the current model fits the proposed data. Signatures that fit the student-t bimodal distribution are kept for survival analysis since they represent a meaningful separation between patient groups. From the two distributions identified in the model, we define sample groups: samples that have a cell type estimate higher than the upper mean are labeled as “patients-up”, samples that have a cell type estimate lower than the mean of the lower distribution are labeled as “patients-down” (Figure 4B). For signatures that don’t fit the student-t bimodal distribution, the patients are separated by the median. However, in cases where a signature had estimates of zero in more than 50% of the tumor-type samples, all samples with an estimate of zero were assigned to the “patients-down” group and all samples with an estimate above zero were assigned to the “patients-up” group. A signature was excluded from survival analysis in a tumor type if less than 10 samples had an estimate above zero.

#### Survival analysis of single cell-type signatures with patient outcomes

TCGA survival information was downloaded from the Xena portal.^67^ We used progression-free interval (PFI) to measure disease progression, except for Acute Myeloid Leukemia (LAML) patients, which only have Overall Survival (OS) available.

To measure the separation between the two sample groups, we used the R package “survminer” for Kaplan-Meier survival analysis, and applied Cox proportional hazards (CoxPH) model^68^ by using R package “survival”.^69^ Reported hazard ratios (HR) were extracted from the CoxPH model and all *p*-values for survival analysis were, unless stated otherwise, *p*-values of the log rank test. We report the results for a 'naive' signature outcome separation (SOS), which is a univariate CoxPH model.

In Table S3 we curated subtype annotations for all TCGA tumor types, mostly from the TCGA PanCanAtlas project^21^ and TCGAbiolinks,^70^ except for DLBCL (diffuse large B-cell lymphoma), which had no subtype information available. Subtype information was used as a covariate in multivariate CoxPH models per tumor type in order to correct a potential imbalance in subtypes, and avoid recapitulating known cancer subtypes by the separation of the patients groups.

To understand how the 217 cell type signatures separate the patients survival, we used Benjamini Hochberg multi-test corrected *p* values from the survival analysis, and focused on the ones that have corrected *p* value lower than 0.05. We also extracted hazard ratio from the models. A hazard ratio greater than 1 indicates the hazard increases and thus the length of survival decreases. When a hazard ratio is smaller than 1, it indicates the cell type variant positively influences the patients’ survival length.

#### Survival analysis of TCT map clusters

We applied survival analysis on TCT map cluster groupings of the TCGA samples analogously to the approach we described previously. First, we analyzed survival in the context of each disease. We defined the main clusters of each disease as any cluster containing 5 or more samples of that disease. Then, we applied CoxPH models between pairs of sample clusters of the same disease, comparing each cluster to the largest cluster, i.e., the cluster with the most samples of that disease. We again provided the disease subtype information curated in Table S3 to the CoxPH models as a covariate in order to correct for a potential imbalance in subtypes. Second, we repeated the same survival analysis in each disease subtype, eliminating the need to provide a subtype covariate and determining which subtypes contributed to the overall findings per disease. The disease and subtype level results are presented in Table S4.

Additionally, Table S4 lists the most differential exemplars between each cluster and the largest cluster in each disease and each subtype. We determined the three highest, and the three lowest exemplars in each comparison using a Student’s t test.

### Additional resources

An interactive browsing session of the TCT map is available online through the UCSC TumorMap portal at bit.ly/TCTmap_217exemplars. The interactive map includes attributes for browsing various results of our analysis including exemplar estimates, TCGA disease categories, TCGA disease subtype categories (Table S5), and TCGA PancanAtlas clustering solutions.^21^

## Supplemental information

Document S1. Figures S1‒S10

Table S1. Single Cell Expression Atlas (SCEA) dataset names, related to Figure 1 Single Cell Expression Atlas (SCEA) datasets used in this paper. The columns show dataset IDs, dataset short and full names, URL link, and publications (if available).

Table S2. Exemplar signature annotation, related to Figure 3 Exemplar signature annotations, short names, tissue of origin, and disease condition. The percentage shows the percent cells in each exemplar signature annotated as a certain cell type/tissue/disease. The annotation comes from two sources: authors annotation or/and inferred annotations based on PanglaoDB. The second tab in this table shows the enrichment analysis results using gene sets curated using PanglaoDB marker genes for cell types.

Table S3. Survival separation of exemplars, related to Figure 5 The table includes numbers and acronyms used in survival analysis.

Table S4. Survival in tumor cell-type (TCT) map clusters, related to Figure 6 Survival analysis results from clusters defined by the TCT map.

Table S5. Subtype definitions, related to Figures 5 and 6 Summary of TCGA tumor subtypes used in this manuscript, including names, number of samples, source, and description.

Table S6. Combined cell-type markers for SCEA annotation, related to Figure 3 Cell-type marker genes used for exemplar signature annotation.

Table S7. Embryonic signatures, related to Figures 3 and 5 Exemplar signatures derived from human embryonic tissues or human embryonic stem cell lines.

Document S2. Article plus supplemental information
