# Supplementary material for: Single-cell signatures identify microenvironment factors in tumors associated with patient outcomes
Source: Cell Rep Methods. 2024 Jun 17;4(6):100799. doi: 10.1016/j.crmeth.2024.100799 (PMC11228369; doi:10.1016/j.crmeth.2024.100799)
Supplement: Document S1. Figures S1‒S10 [file mmc1.pdf]

Cell Reports Methods, Volume *4*

Supplemental information

Single-cell signatures identify microenvironment factors in tumors associated with patient outcomes

Yuanqing Xue, Verena Friedl, Hongxu Ding, Christopher K. Wong, and Joshua M. Stuart

Cell Reports Methods

Supplemental information

Single-cell signatures identify microenvironment factors in tumors associated with patient outcomes

Yuanqing Xue, Verena Friedl, Hongxu Ding, Christopher K. Wong, Joshua M. Stuart

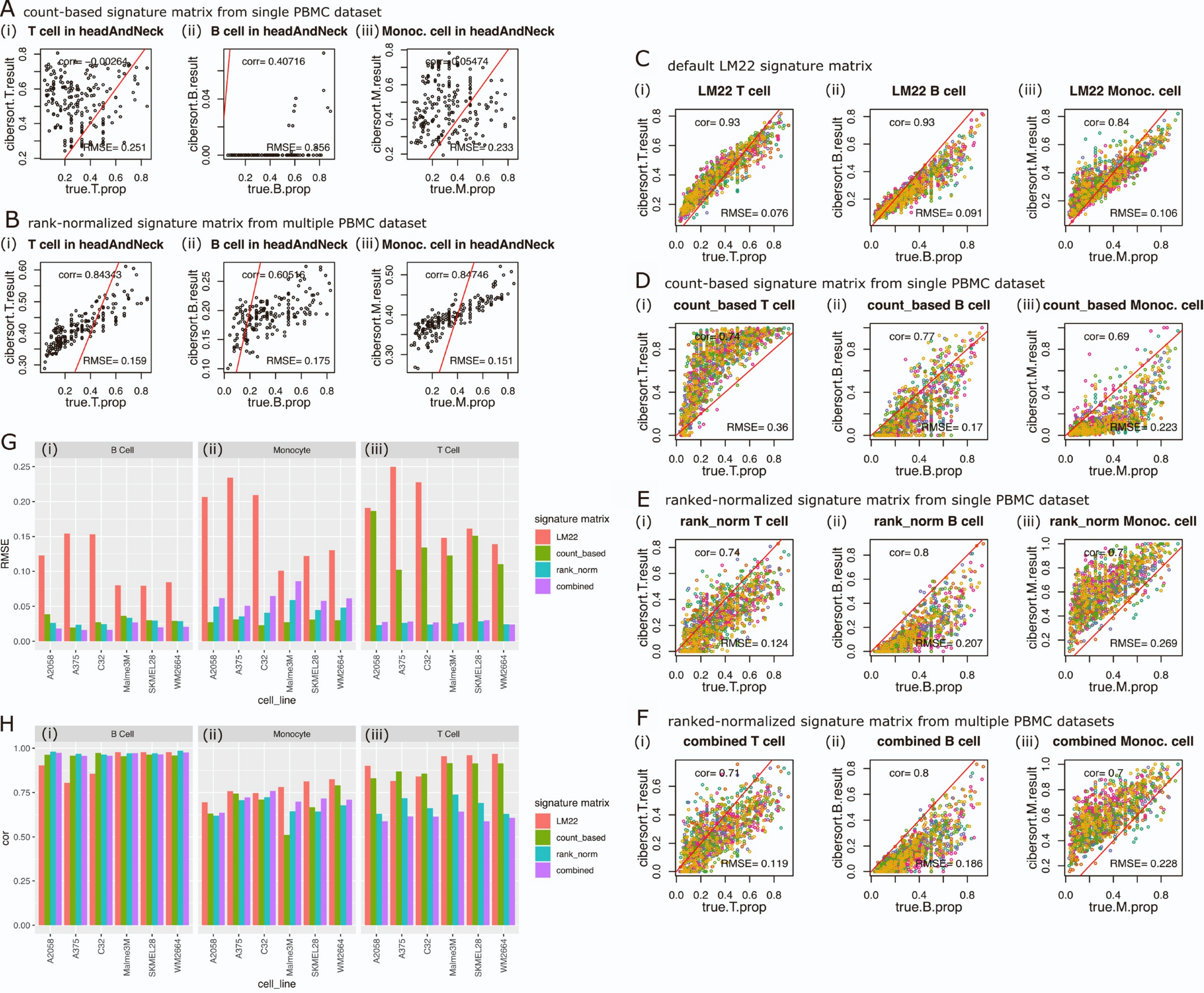

Figure S1. Related to main Figure 2. (A-B) Validation of scBeacon workflow in synthetic mixtures of a scRNA-Seq head and neck cancer dataset. (A) Correlation between the true mixture proportions in synthetic mixtures for (i) T-cells, (ii) B-cells, and (iii) Monocytes from deconvolution of a scRNA-Seq head and neck cancer dataset using a count-based signature matrix from a single PBMC scRNA-Seq dataset (10X-v2). Red line marks the correct estimate (x=y). Cell type ratios are normalized to sum up to 1. (B) Same as A, but using a rank-normalized signature matrix from the combination of multiple PBMC scRNA-Seq datasets (all PBMC datasets from Figure 1B-D, except Smart-seq2: 10X chemistry v1-v3, CEL-Seq2, Drop-Seq, inDrops, Seq-Well). (C-F) Validation of scBeacon workflow in synthetic mixtures from sorted bulk RNA-Seq datasets. (C) Correlation between the true mixture proportion in synthetic mixtures from bulk RNA-Seq and the deconvolution results of using Cibersort's default signature matrix, LM22 to estimate the proportions of (i) T cells, (ii) B cells, and (ii) Monocytes. The synthetic mixtures are built from bulk RNA-Seq of six different cancer cell lines (colors) and bulk RNA-Seq of purified immune cells. Red line marks the correct estimate (x=y). Cell type ratios are normalized to sum up to 1. (D) Same as A, but using a count-based signature matrix from a single PBMC scRNA-Seq data set (10X-v2). (E) Same as A, but using a rank-normalized signature matrix from a single PBMC scRNA-Seq data set (10X-v2). (F) Same as A, but using a rank-normalized signature matrix from the combination of multiple PBMC scRNA-Seq data sets (all PBMC data sets from Figure 1B-D, except Smart-seq2: 10X chemistry v1-v3, CEL-Seq2, Drop-Seq, inDrops, Seq-Well). (G-H) Overview of validation of scBeacon workflow in synthetic mixtures from sorted bulk RNA-Seq datasets. (G) RMSE between the true mixture proportion in synthetic mixtures from bulk RNA-Seq and the deconvolution results of using Cibersort's default signature matrix, LM22, and summing more specific deconvolution results into (i) B cells, (ii) Monocytes, and (iii) T-cells. The synthetic mixtures are built from bulk RNA-Seq of six different cancer cell lines (colors) and bulk RNA-Seq of purified immune cells. For each cell line (columns) and for each signature matrix (colors) plotted in Figure S3. (H) Same as A, but shows Pearson correlation coefficients for each cell line (columns) and for each signature matrix (colors) plotted in Figure S3.

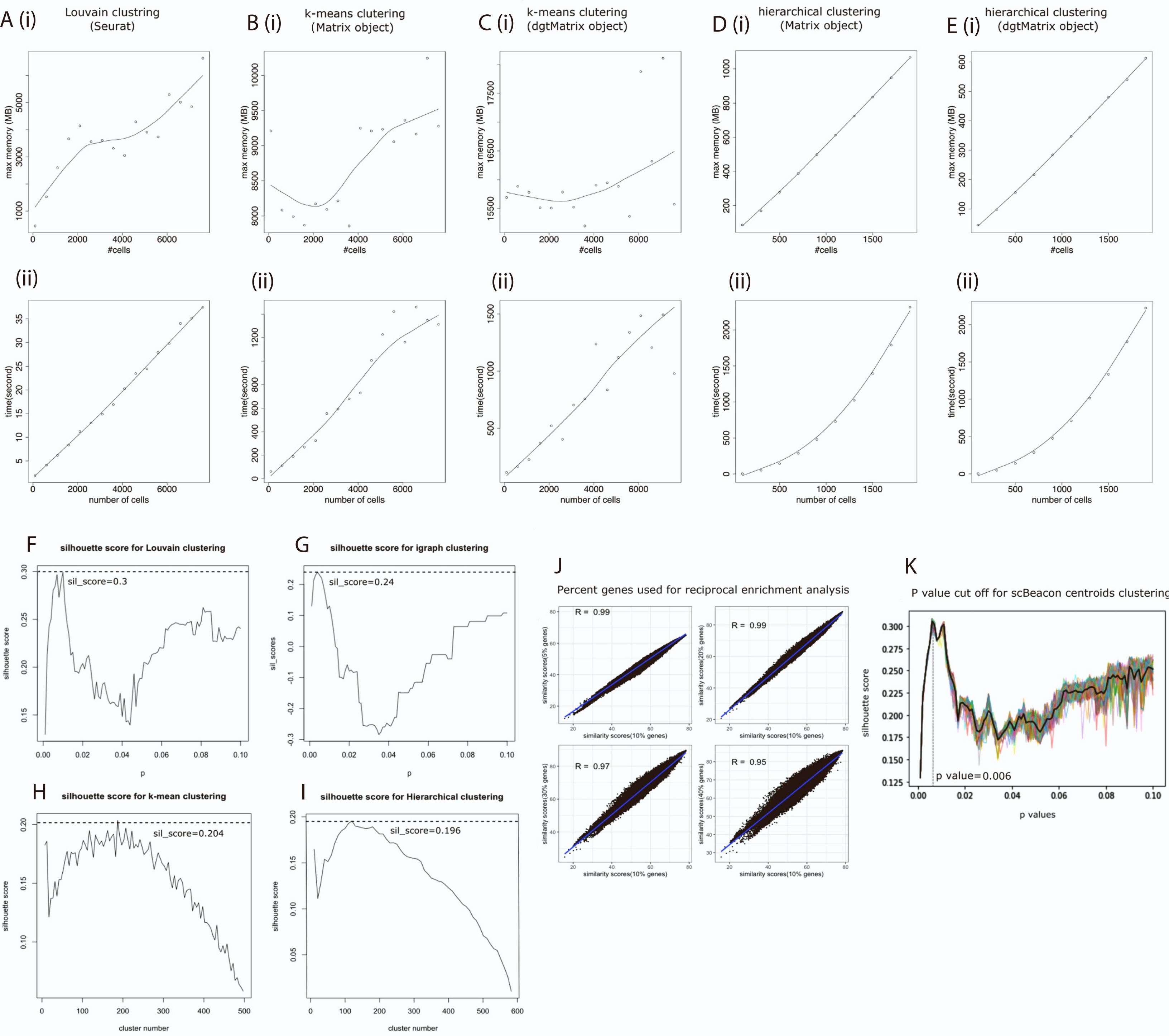

Figure S2. Related to main Figures 1 and 3. (A-E) Comparison of computation cost for different clustering algorithms Comparing computation cost in clustering algorithms. (A) Louvain(from Seurat package), (B) K-mean(using Matrix object), (C) K-mean(using dgtMatrix), (D) hierarchical(using Matrix object) and (E) hierarchical(using dgtMatrix) clustering. On each plot, the y-axis shows the memory usage (i) and computing speed (ii), plotted against the number of cells in a prototypical scRNA-Seq dataset (x-axis) used for benchmarking clustering. (F-I) Use silhouette score to decide the best clustering method for scBeacon centroids. To choose the optimal clustering method for scBeacon centroids, we tested four most used clustering algorithms, (F) Louvain clustering, (G) igraph clustering, (H) hierarchical clustering and (I) k-mean clustering, and compared the highest silhouette scores in each algorithm. Louvain clustering reached the highest silhouette score, at 0.3, generating 163 clusters. (J-K) Clustering solution and percent genes for reciprocal enrichment analysis in scBeacon. (J) We chose 10% top ranked genes to perform reciprocal enrichment analysis, here we compared the similarity score generated using top 5%, 20% 30% and 40% genes using correlation plot, with correlation coefficient shown in the plots. (K) Use silhouette score to decide p-value cut-off to build scBeacon centroids adjacency matrix for louvain clustering (resolution=0.7). To decide scBeacon centroids clustering solution, we evenly spaced 100 p values from 0 to 0.1 and calculated silhouette scores for louvain clustering. For each p-value, silhouette scores were calculated under 50 different random seeds, the black line is the average silhouette score of the 50 silhouette scores calculated. From the plot, the highest silhouette score is reached at p value=0.006, silhouette score=0.3, generating 217 clusters.

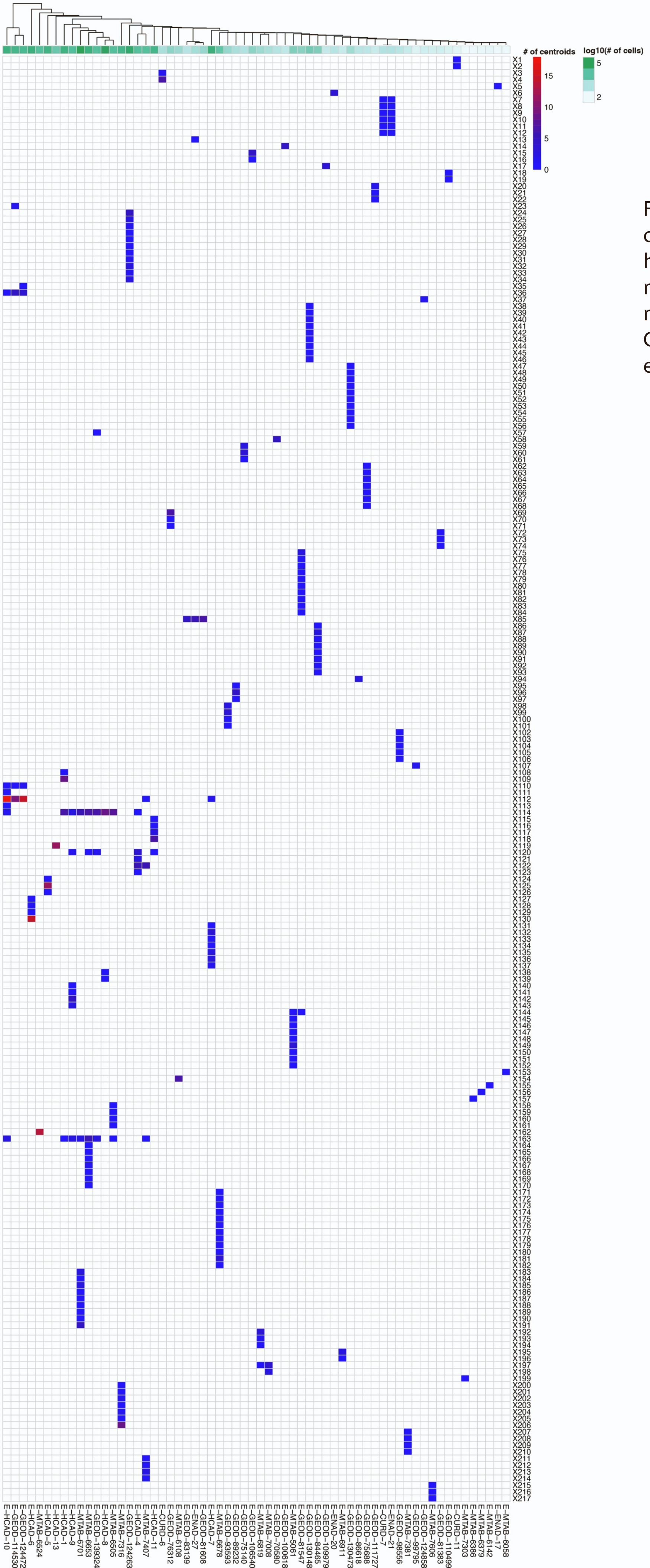

Figure S3. Related to main Figure 3. SCEA dataset origin for scBeacon meta-cluster exemplars. The heatmap color shows the number of centroids that are merged into meta-clusters. The number of cells measured in each dataset is annotated on top. Columns are SCEA datasets and rows are meta-cluster exemplars.

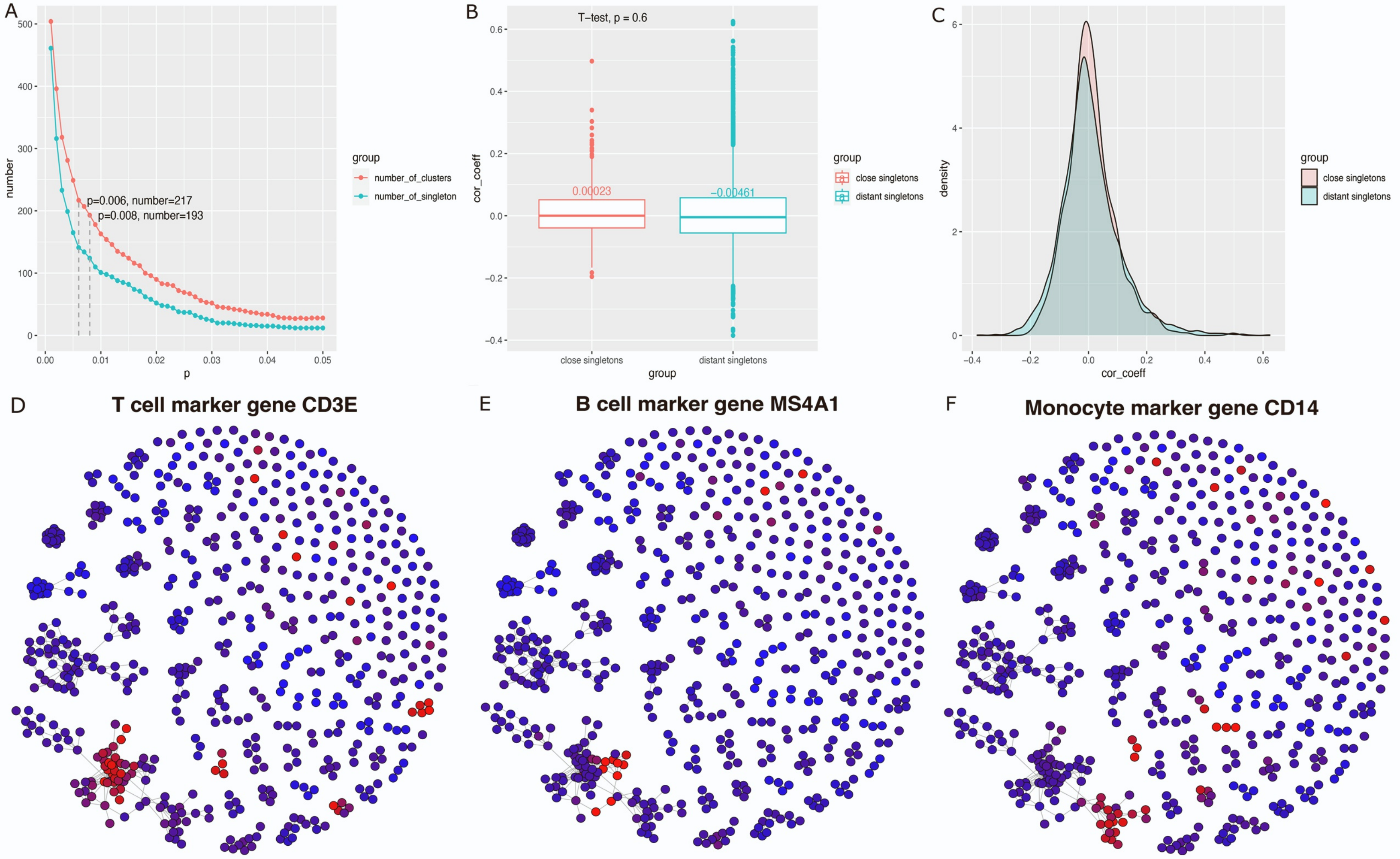

Figure S4. Related to main Figure 3. (A-C) The subset of SCEA 217 signatures that are "singletons," i.e. made up of one cluster in a metacluster, reflect distinct signatures that should not be merged with others as "close" versus "distant" singletons have a similar distribution in correlations of Cibersort inferences across TCGA. (A) The number of clusters (y-axis) and singletons change as a function of the similarity measurement threshold p (x-axis). The higher p, the higher the similarity level between the cluster centroids, generating fewer exemplars and singletons. Vertical lines indicate the cutoff used for the manuscript results (p=0.006 resulting in 217 metaclusters) and the second cutoff (p=0.008) was drawn to identify "close" versus "distant" singletons; i.e. close singletons defined as being those merged into a single cluster at p=0.008 compared to being separate clusters for p=0.006. (B-C) Separate singletons in 217 exemplars into two groups: singletons that are closer and distant in clustering space. The Pearson correlation coefficient was calculated between the Cibersort inference vectors of two singleton exemplars. Close versus distant singletons yielded a similar distribution in Pearson correlation coefficients illustrated as either box plots (B) or as overlapping histograms (C), consistent with maintaining the singletons as separate exemplars as was done for the analysis in the manuscript. (D-F) Immunomarkers in scBeacon clusters. scBeacon centroids(colored circles) colored by (D) T cell(CD3E), (E) B cell(MS4A1) and (F) monocyte(CD14) marker genes (low expression, blue; high expression, red).

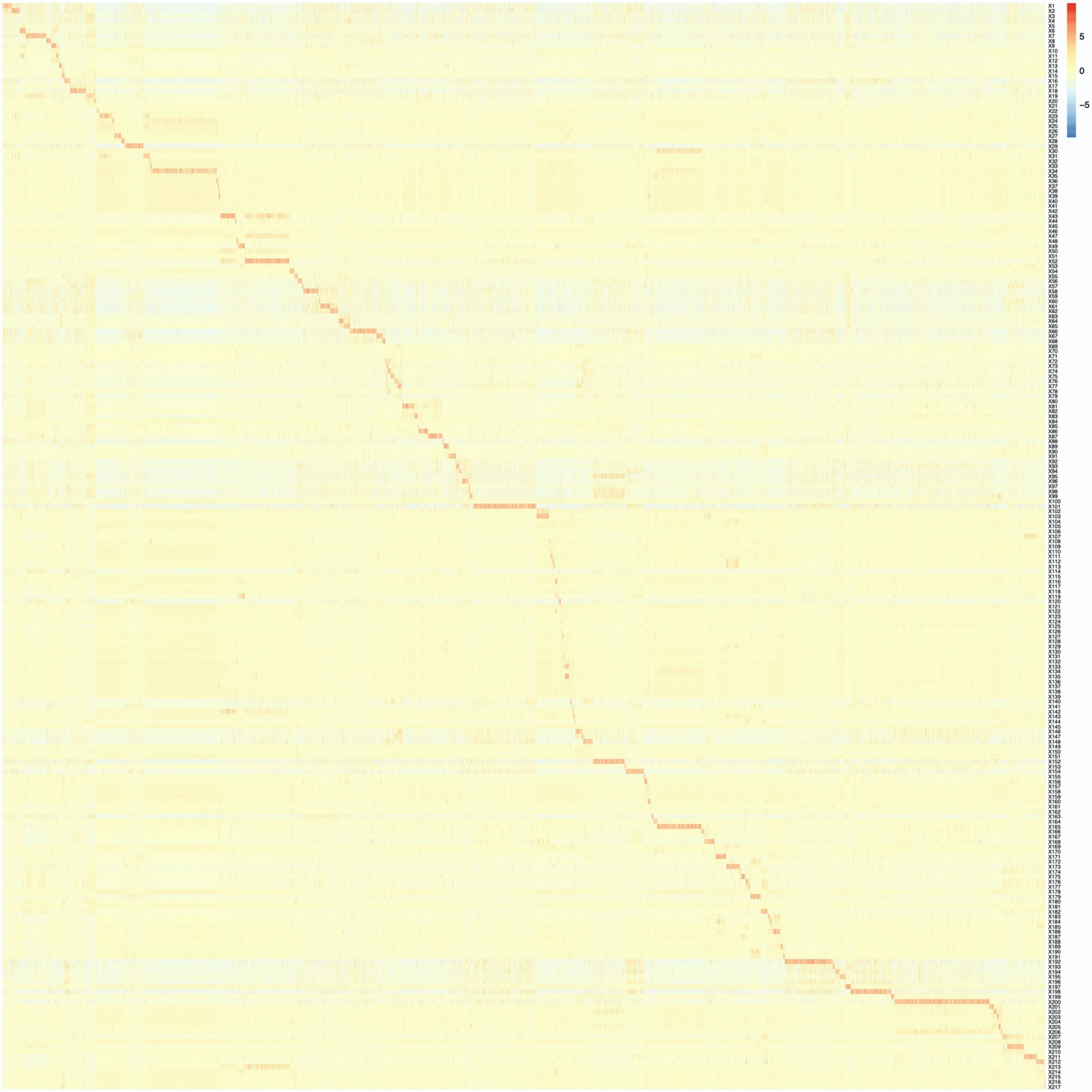

Figure S5. Related to main Figure 3. Signature matrix for meta-cluster exemplars. The heatmap shows the average gene expression in the selected 3988 signature genes. The middle redline shows the differential expressed genes for each exemplar that separate it from others.

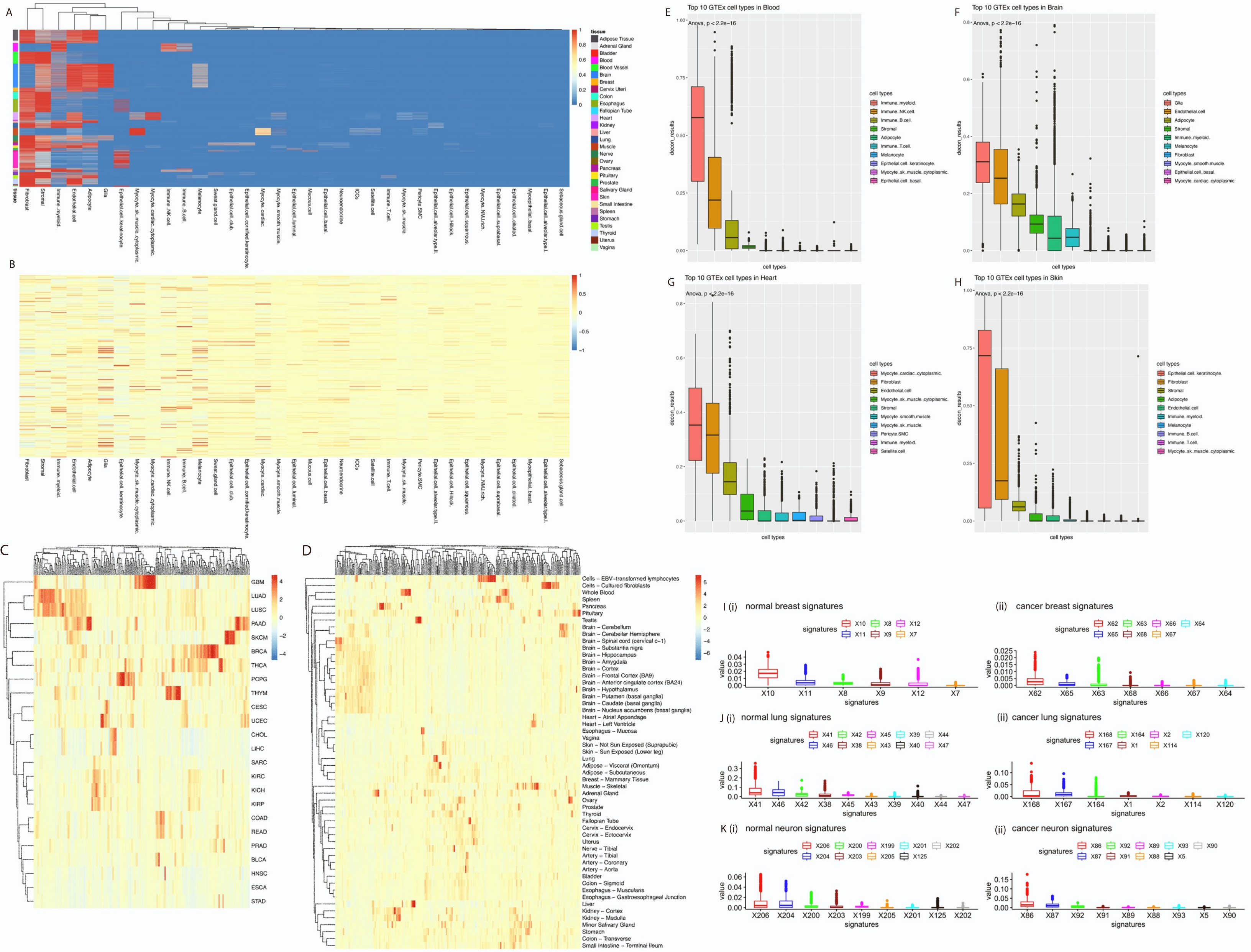

Figure S6. Related to main Figure 4. (A-F) Comparison of scBeacon deconvolution using GTEx snRNA-seq signatures to SCEA-derived 217 signatures, to infer cell types in bulk GTEx normal RNA-seq data. (A) Overview of deconvolution of bulk samples from the GTEx Consortium dataset 23 in which cell type signatures were derived from the GTEx single-nucleus RNA sequencing dataset 22, cell type annotation for single cells, and tissue annotation for bulk samples were taken from curated metadata. (B) Pearson correlation between GTEx snRNA-seq deconvolution results and SCEA-derived 217 deconvolution results on GTEx bulk samples. With rows as SCEA-217 signatures and columns as GTEx snRNA-seq cell type signatures. (C-D) Normal tissue deconvolution analysis. (C) CIBERSORT estimation of 217 exemplars on the normal bulk samples in the TCGA collection. Entries of the matrix show the the Cibersort estimation for one of the 217 exemplars (columns) averaged across the samples within one of 24 TCGA cancer cohorts (rows). (D) CIBERSORT estimation of 217 exemplars (columns) averaged across GTEx samples having the same tissue type annotation (rows). (E-H) The top 10 GTEx cell type signatures by Cibersort scores when deconvoluting GTex bulk tissues of (E) blood, (F) brain, (G) heart, and (H) skin. (I-K) Tissue specific signatures estimation in matching cancer and normal samples. The barplot shows the average CIBERSORT estimation in signatures derived from normal (left) and cancer (right) tissues. (I)(i) normal breast signatures in normal breast samples. (I)(ii) cancer breast signatures in breast cancer samples. (J)(i) normal lung signatures in normal lung samples. (J)(ii) cancer lung signatures in lung cancer samples. (K)(i) normal neuron signatures in normal brain samples. (K)(ii) cancer neuron signatures in brain tumor samples. The signatures in each barplot are ordered by the average CIBERSORT estimation values, from highest to lowest.

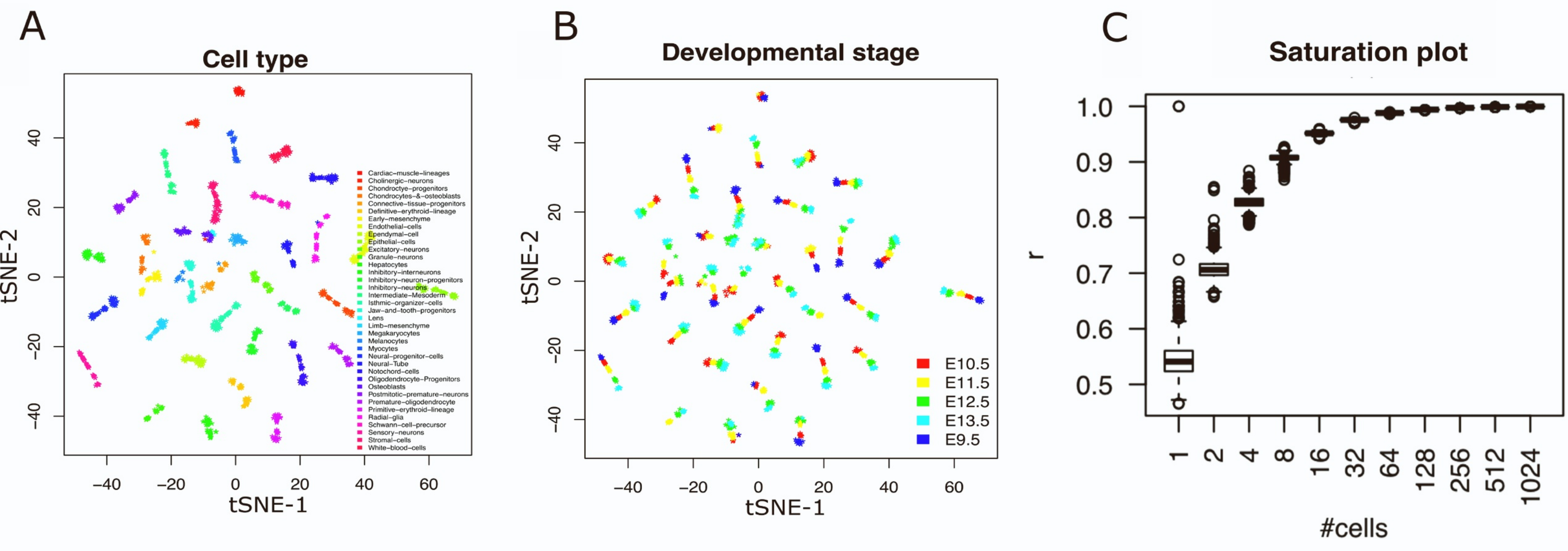

Figure S7. Related to main Figures 1 and 3. (A-B) tSNE plot of Mouse Organogenesis Cell Atlas (MOCA) dataset after scBeacon pipeline. Clustering solution is curated by cell type annotation in MOCA. Data points are centroids. (A) tSNE plot of MOCA centroids, colored by cell types. (B) same as A, colored by mouse developmental stages from E9.5 to E13.5. (C) Number of cells needed to reliably estimate cluster centroid. Y-axis shows Spearman correlation coefficient between the centroid from all the cells of a cluster with centroid from sampled cell, number of cells sampled shows on x-axis. Correlation coefficient saturates when samples of over 50 cells are included in the analysis.

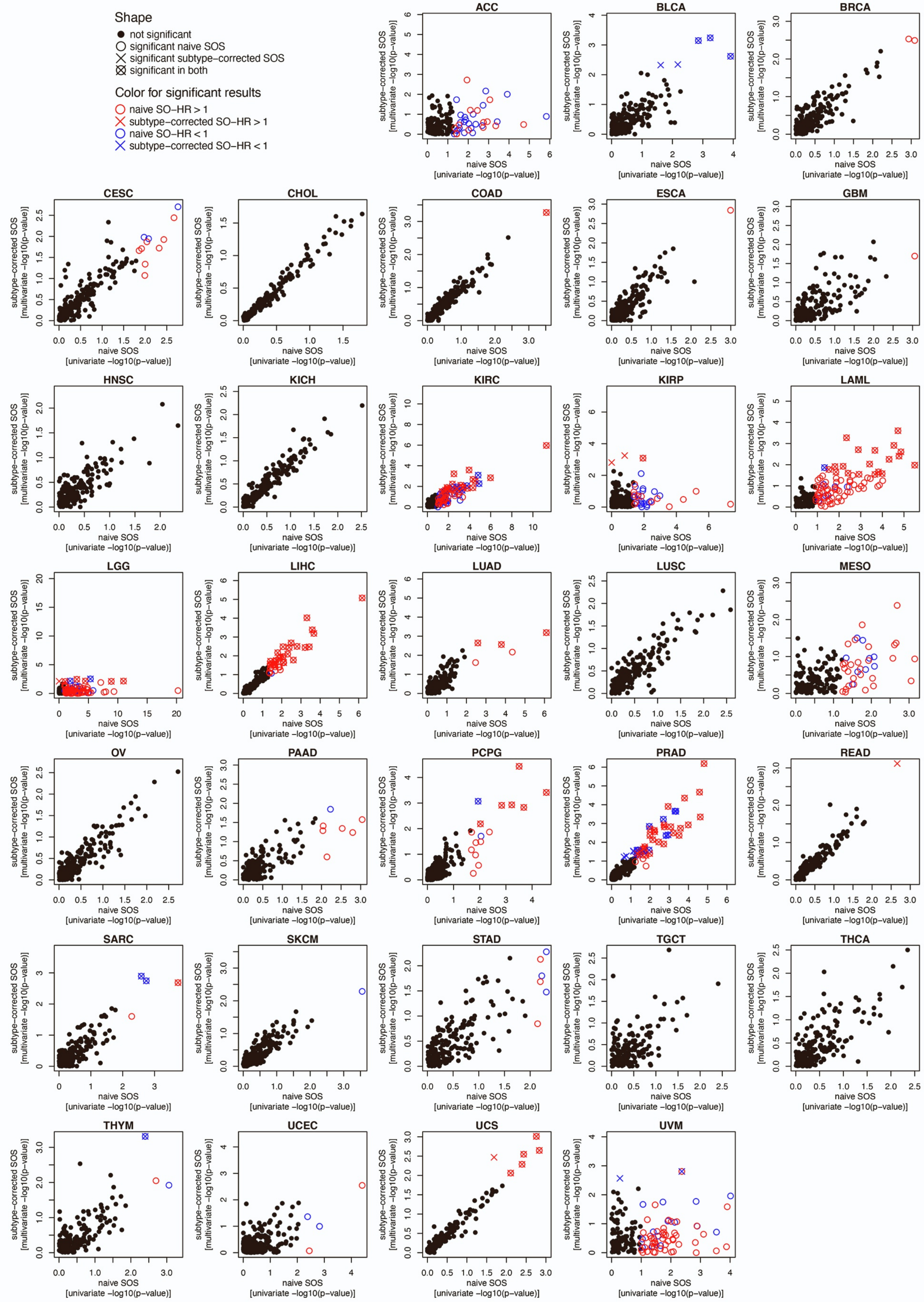

Figure S8. Related to main Figure 5. Signature outcome separation (SOS) in all TCGA tumor types. Plots show -log10(p-value) (not multiple-testing corrected) from the CoxPH survival model between 'up' and 'down' sample groups for each signature that passes the bimodal test in the respective tumor type. The results from univariate CoxPH models are on the x-axis, the results from multivariate CoxPH models, correcting for unbalanced cancer subtypes, are on the y-axis. Results are considered significant with an FDR-corrected p-value < 0.25. Survival models which show significant separation with a signature outcome hazard ratio (SO-HR) above 1 are marked in red ('up'-group has a worse chance of survival than 'down'-group). Survival models which show significant separation with a SO-HR below 1 are marked in blue ('up'-group has a better chance of survival). Outcome is measured in PFI (progression free interval) for all tumor types, except for LAML which only has OS (overall survival) available.

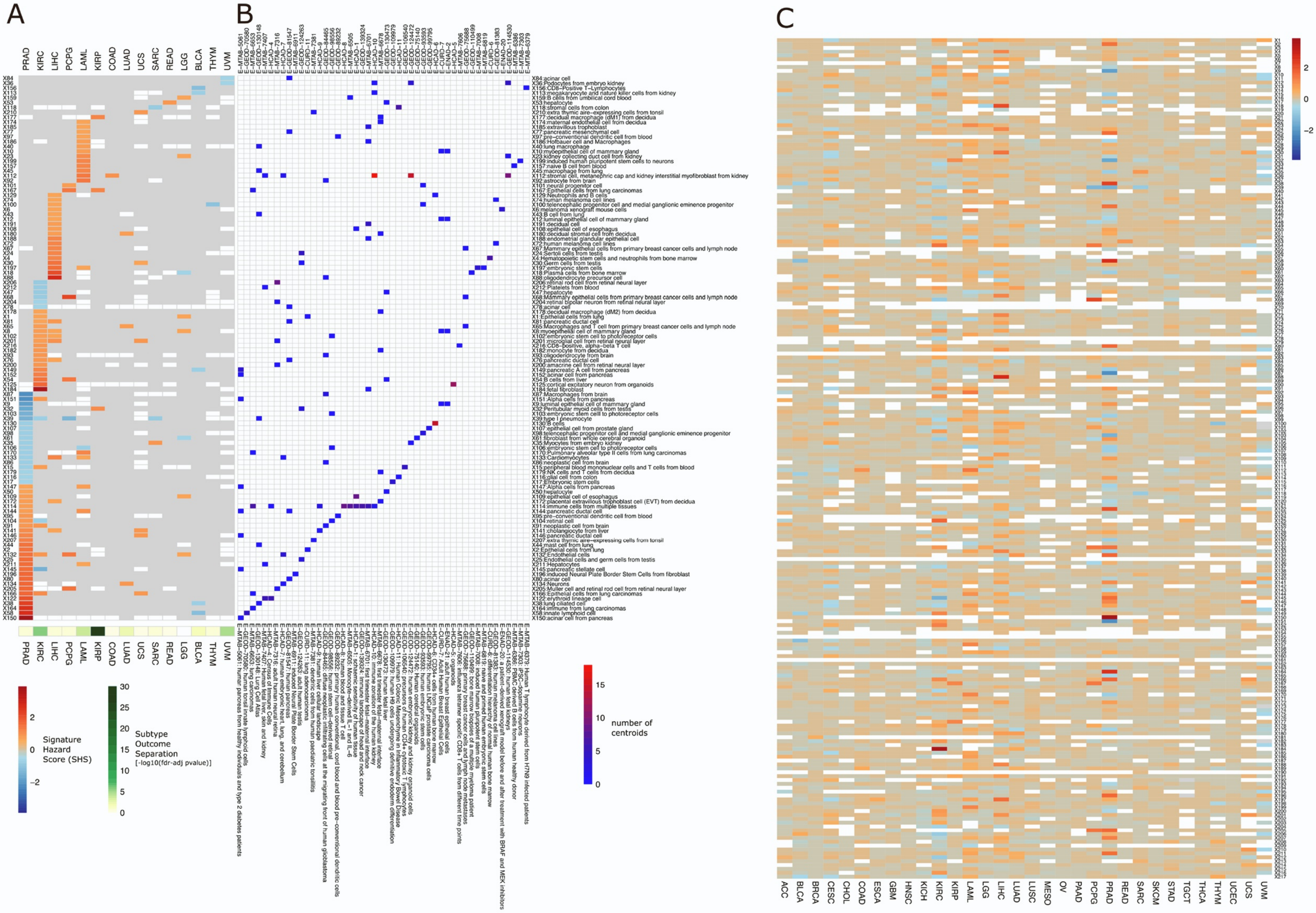

Figure S9. Related to main Figure 5. (A-B) Overview of patient outcomes implicated by individual cell types in several tumor types. (A) Significant association with patient outcome, after accounting for published subtypes, was found for 112 (out of the starting 217) cell type exemplars (rows) in at least one of 15 TCGA tumor type cohorts (columns). Entries in the map illustrate either poorer (red) or better (blue) outcomes as determined by fitting the subtype-corrected results (log10 of the FDR-corrected P-value) from the Cox Proportional Hazards model (CoxPH). All (non-significant) results with an FDR-adjusted P-value > 0.25 are shown in gray. Results in white were excluded from survival analysis because the exemplar deconvolution estimates in the tumor type failed the bimodality test and they were detected in less than 10 samples. Full results can be found in Figure S8. For each tumor type, the bottom row plots the results of training a regression model that used all 217 exemplars to predict outcomes in each cohort with darker green highlighting tumor types with higher significance. (B) Annotation of the exemplars from part A to show what EBI SCEA datasets contributed to their definitions (columns) and a short description of the exemplar determined from author-provided statements or marker gene enrichment analysis (see Methods and full annotation list in Table S2). (C) Signature Hazard scores. The heatmap shows the Signature Hazard Score (SHS) which is defined as log10(fdr-corrected p-value) from the multivariate CoxPH model (correcting for covariate subtype) between 'up' and 'down' sample groups as defined by the bimodal distribution test or median split. Negative values in blue represent a low SHS for which the 'up' group has a significantly better chance of survival than the 'down'-group (low hazard if signature is detected). Positive values in red mark cases in which the 'up'-group has a worse chance of survival (high hazard if signature is detected). Signatures in white are signatures that were not used for survival analysis because at least one of the sample groups was too small (<10 samples).

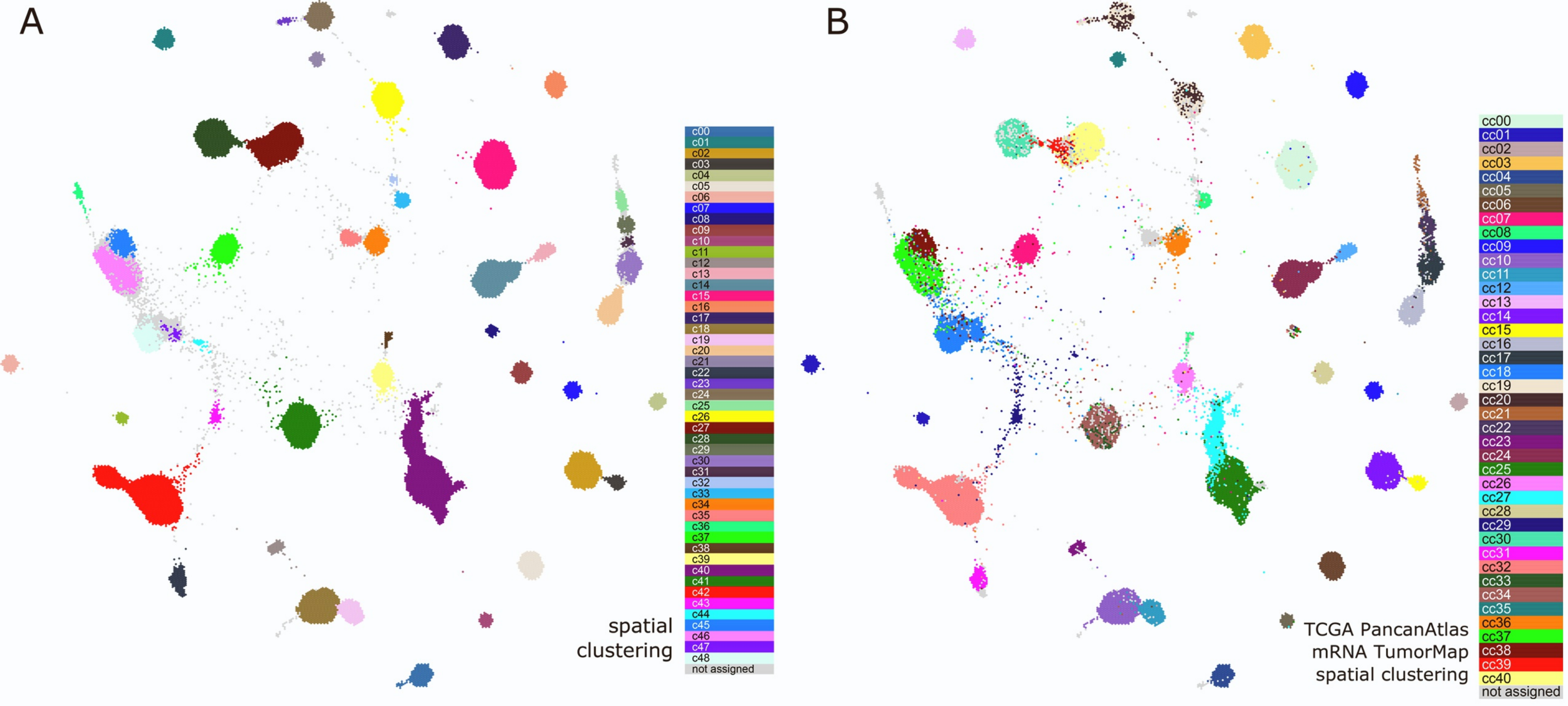

Figure S10. Related to main Figure 6. (A) Tumor Cell-Type (TCT) Map in which the dots represent tumor samples positioned by their microenvironment content as captured in vectors representing the CIBERSORT estimated levels for each of the 217 exemplar signatures in each tumor sample. Samples were clustered into one of 48 different clusters (colors; hdbscan with parameter minimal cluster size set to 20). (B) Tumor Cell-Type (TCT) Map in which the dots represent tumor samples positioned by their microenvironment content as captured in vectors representing the CIBERSORT estimated levels for each of the 217 exemplar signatures in each tumor sample. The colors represent the sample clustering solution from TCGA PanCan Atlas tumormap based on mRNA gene expression data.
